# Exploring the Redox Properties of the Low-Miller Index Surfaces of Copper Tungstate (CuWO_4_): Evaluating the Impact of the Environmental Conditions on the Water Splitting and Carbon Dioxide Reduction Processes

**DOI:** 10.1021/acs.jpcc.3c04413

**Published:** 2023-09-15

**Authors:** Xuan Chu, David Santos-Carballal, Nora H. de Leeuw

**Affiliations:** † School of Chemistry, University of Leeds, Leeds LS2 9JT, U.K.; ‡ Department of Earth Sciences, Utrecht University, Princetonplein 8A, Utrecht 3584 CD, The Netherlands

## Abstract

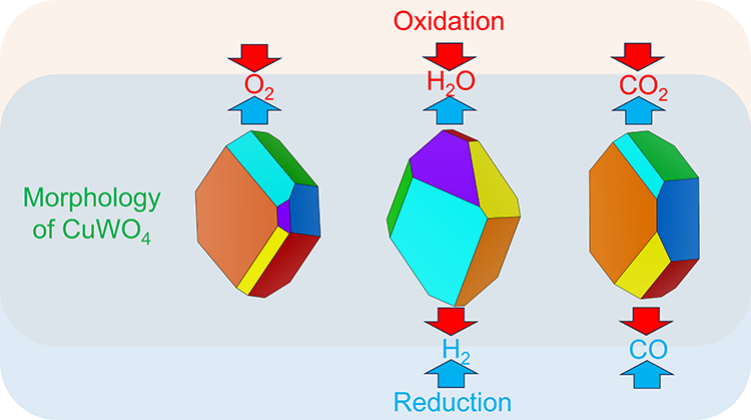

Photocatalysis has gained significant attention and interest as an environmentally friendly and sustainable approach to the production of hydrogen through water splitting and the reduction and conversion of CO_2_. Copper tungstate (CuWO_4_) is a highly promising candidate for these applications owing to its appropriate bandgap and superior stability under different conditions. However, the redox behavior of the CuWO_4_ surfaces under different environments and their impact on the morphology of the material nanoparticles, as well as the electronic properties, remain poorly understood. In this study, we have employed density functional theory calculations to investigate the properties of the bulk and pristine surfaces of CuWO_4_ and how the latter are impacted by oxygen chemisorption under the conditions required for photocatalytic water splitting and carbon dioxide reduction processes. We have calculated the lattice parameters and electronic properties of the bulk phase, as well as the surface energies of all possible nonpolar, stoichiometric, and symmetric terminations of the seven low-Miller index surfaces and found that the (010) and (110) facets are the thermodynamically most stable. The surface-phase diagrams were used to derive the equilibrium crystal morphologies, which show that the pristine (010) surface is prominent under synthesis and room conditions. Our crystal morphologies suggest that the partially oxidized (110) surface and the partially reduced (011) surface may play an important role in the photocatalytic splitting of water and CO_2_ conversion, respectively. Our results provide a comprehensive understanding of the CuWO_4_ surfaces under the conditions of important photocatalytic applications.

## 1 Introduction

In recent decades, the shift toward carbon-free, low-cost energy production has prompted significant interest in the use of semiconductors in photoelectrochemical (PCE) processes to store energy in chemical bonds.^1–3^ Although requiring the input of energy, one promising method to obtain clean energy is through the water splitting reaction, which generates molecular oxygen and hydrogen, which are an oxidizing agent and a fuel, respectively.^4^ Photocatalysis offers an attractive route by using solar energy to generate hydrogen, satisfying both environmental and economic standards for green energy sources.^5,6^

Several materials, such as bismuth vanadate,^7^ tungsten diselenide (WSe_2_),^8^ porous organic polymers,^9^ metal compounds,^10^ and binary metal oxides, for example, ZnO,^10^ TiO_2_,^11^ Fe_2_O_3_,^12^ and WO_3_,^13^ have been studied as catalysts for water splitting because of their low cost and high chemical stability. However, their applications have been limited due to inherent flaws, including inadequate light adsorption, large bandgap, low mobility of the charge carriers, and short hole diffusion length. The main reason for these shortcomings is that the valence bands of most binary metal oxides have strong O 2p orbital characteristic, which lies below the water splitting potential (1.23 V at 25 °C and 1 atm).^14,15^

In contrast, ternary metal oxides, such as BiVO_4_,^16^ ZnWO_4_,^17^ and CuWO_4_,^18–20^ have been shown to perform better than binary metal oxides in photocatalytic water splitting owing to the contribution by the metal d orbital and O 2p orbital to the valence band maxima.^14^ In particular, copper tungstate (CuWO_4_), which is an n-type semiconductor, has attracted increasing interest for the photocatalytic water splitting process^18–26^ owing to its appropriate bandgap of 1.6–2.4 eV, eco-friendliness, low cost, nontoxicity, and high stability under various conditions.

Semiconducting materials, such as zinc oxide (ZnO) and cadmium sulfide (CdS), have been proposed for photocatalytic CO_2_ reduction, but their ability to absorb only a narrow range of light wavelengths and their inefficient transfer of the photogenerated electrons make them unsuitable candidates for this process. CuWO_4_ has also shown potential in photocatalytic CO_2_ reduction, which is an important step for carbon capture, with high selectivity to specific chemicals, such as methanol and other oxygenated species.^27,28^

A number of experimental and computational studies have provided a relatively comprehensive understanding of the crystal morphology, physicochemical properties, synthesis methods, modification strategies, and PCE applications of CuWO_4_.^18,19,23–26,29,30^ However, the redox behavior of the low-Miller index surfaces of CuWO_4_ under different environment-dependent conditions and its impact on the stability, structure, and electronic properties of the surfaces and on the crystal morphology of this material remain unknown. In this work, we have employed calculations based on the density functional theory (DFT) to study the structural and electronic properties of the bulk phase of CuWO_4_, as well as all nonpolar, stoichiometric, and symmetric terminations of its low-Miller index surfaces. We discuss (i) the redox properties of the thermodynamically most stable surface terminations as a function of the temperature and the partial pressure of oxygenated species, (ii) the morphology of the nanocrystals of CuWO_4_ under synthesis conditions, and (iii) the typical industrial conditions for photocatalytic water splitting and CO_2_ conversion. This computational study aims to provide guidance for future research in utilizing CuWO_4_ for photocatalytic water splitting and CO_2_ reduction processes.

## 2 Computational Methods

### 2.1 DFT Calculations

DFT calculations were performed using the Vienna Ab Initio Simulation Package (VASP) within the projector augmented wave method.^31–33^ We employed the Perdew–Burke–Ernzerhof functional to treat the exchange–correlation energy^34^ and the projector augmented wave formalism to handle the core states of Cu:[Ar], W:[Xe], O:[He], and C:[He], as well as their interactions with the valence orbitals; the 1s state of the H atom was treated as valence. The D3 method with the Becke–Johnson damping^35^ was used to model the long-range van der Waals interactions. Periodic plane wave basis sets were used to expand the Kohn–Sham valence states with a cutoff energy of 400 eV. All calculations were spin-polarized, with the magnetic moments set to a high spin antiferromagnetic alignment in the Cu atoms in the alternating layers along the [001] direction. The DFT + *U* method^36^ was used to account for the electronic self-interaction and to improve the description of the electronic structure. The on-site Coulomb interaction term^37^ was tested between 3 and 10 eV for both Cu and W, and we found that when *U*_eff_ = 7.5 eV for Cu, we obtain the best description of the lattice parameters and the bandgap of CuWO_4_ with respect to the experimental results. Our tests indicated that the *U* correction is not needed for the W atoms as they lose all their d electrons upon formation of CuWO_4_. For the calculations of the bulk material, we used a triclinic primitive unit cell containing 12 atoms (Cu_2_W_2_O_8_), which was integrated in the reciprocal space with a regular Γ-centered 4 × 3 × 4 mesh of *k*-points.^38^ We used the conjugate gradient algorithm^39^ to fully relax all structures until the forces on each atom were less than 0.01 eV/Å and the energy difference between consecutive self-consistent loop steps was below 1 × 10^–6^ eV.

### 2.2 Surface Energy Diagrams

We have used the dipole method proposed by Tasker^40^ to construct the surface slab models for CuWO_4_. This method considers the crystal as a stack of planes and ensures that no dipole moment or surface charge exists perpendicular to the surface. The dipole method for polar solids considers three types of surfaces. In type 1, each plane has no net charge as they are composed of cations and anions in stoichiometric ratio, which makes the surface nonpolar. In type 2, the dipole moment between charged planes is canceled within the three-layer symmetric stacking sequence containing an integer number of formula units. In type 3, there is a dipole moment perpendicular to the surface due to the nonsymmetrical stacking of alternating charged planes forming an integer number of formula units. We reconstructed such terminations into nonpolar surfaces by moving half of the ions with the same charge from the top to the bottom of the slabs. The dipole method also ensures that the electrical field vanishes within the surface slab, and the potential at each ion site becomes identical to the constant bulk value.

We have modeled the isolated molecules, including O_2_ in the triplet state, H_2_, H_2_O, CO, and CO_2_, in an 8 × 8 × 8 Å^3^ periodic box, where only the Γ-point^38^ was sampled.

An improved grid-based algorithm was used to obtain the effective atomic Bader charges^41–43^ and the atomic magnetic moments. The work function, defined as the minimum thermodynamic energy required to remove one electron from the Fermi level (*E*_F_) at the CuWO_4_ surface to the vacuum level (*E*_vac_), is calculated using the following equation^44^
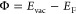
1

To create all seven surfaces, we employed the dipole method as implemented in the METADISE package.^45^ To model the pristine as well as the partially reduced and partially oxidized surfaces, we found that the surface energy converges when the bottom 25% of the atomic layers were fixed at their relaxed bulk positions, whereas the rest of the atomic planes were allowed to relax to obtain a single relaxed surface. We introduced a 10 deep vacuum perpendicular to the surface to create a slab of the material. To sample the Brillouin zone, we tested and applied different Monkhorst–Pack meshes^38^ of Γ-centered *k*-points depending on the low-Miller index surface. For the (001), (011), and (110) surfaces, we used a 4 × 3 × 1 *k*-point mesh, while for the (101) and (111) surfaces, we used a 3 × 3 × 1 *k*-point grid. The (010) surface was sampled using a 4 × 4 × 1 *k*-point mesh, whereas for the (100) surface, we used a 3 × 4 × 1 *k*-point mesh. The tetrahedron method with Blöchl corrections^46^ was used for the geometry optimizations and to obtain accurate energies of the bulk and surfaces.

The surface energies of the slabs before (γ_u_) and after (γ_r_) relaxation are defined as^44^
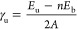
2
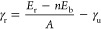
3where *E*_u_, *E*_r_, and *E*_b_ represent the energies of the unrelaxed slab, half-relaxed slab, and one formula unit in the bulk, respectively; *A* represents the surface area; and *n* is the number of formula units in the surface slab. The degree of relaxation is calculated as^44^
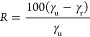
4

The relaxation of the interplanar distance is defined as^44^
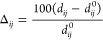
5where *d*_*ij*_ and *d*_*ij*_^0^ represent the distance between the topmost layer *i* and *j* = *i* + 1 in the relaxed surface model and bulk, respectively.

## 3 Results and Discussion

### 3.1 Bulk Properties of CuWO_4_

**Figure 1:**
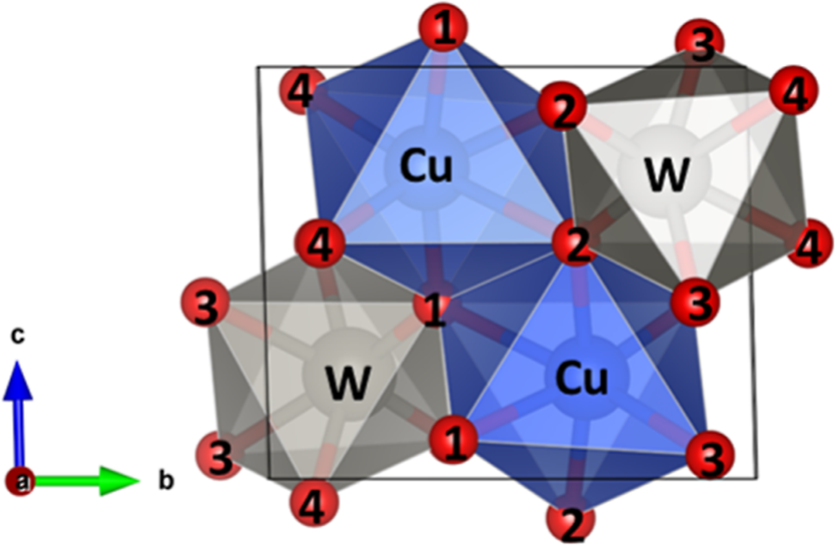
Polyhedral model of triclinic CuWO_4_ containing two formula units. The symmetrically inequivalent O atoms are labeled with numbers. Cu atoms are in blue, W atoms are in gray, and O atoms are in red.

The conventional triclinic unit cell of CuWO_4_, which contains two formula units, is shown in Figure 1. The Cu^2+^ and W^6+^ cations are surrounded by six O^2–^ anions, forming the corner-shared CuO_6_ and WO_6_ octahedra. The Jahn–Teller effect breaks the Cu 3d e_g_ orbital degeneracy, resulting in the triclinic *P*1® symmetry and distortion of the CuO_6_ octahedra. Due to the O sharing, the WO_6_ octahedra also undergo a slight distortion. We found in our optimized structure of the bulk that the cation–oxygen bond lengths range from 1.951 to 2.403 Å in the CuO_6_ octahedra and from 1.792 to 2.192 Å in WO_6_. Figure 1 shows the four inequivalent O atoms within the unit cell of CuWO_4_. O1 and O2 are linked to two Cu^2+^ cations and a single W^6+^ cation, while O3 and O4 are connected to two W^6+^ cations and a single Cu^2+^ cation.

Table 1 summarizes the structural, electronic, and magnetic properties of CuWO_4_. The calculated lattice parameters agree very well with the experimental data, with a deviation of only 1.5%.^47^ The atomic Bader charges for Cu, W, and O are calculated as 1.244, 2.895, and −1.035 e^–^/atom, respectively, that is lower than the formal charges of the ions which is usual in the Bader method.^44^ The calculated magnetic moment for Cu is 0.78 μ_B_ atom^–1^, which corresponds to a high-spin electronic configuration of e_g_^3^ t_2g_^6^. This result is in good agreement with both experiments and previous computational estimates.^48,49^ We observed relatively small magnetic moments of 0.03 μ_B_ atom^–1^ for O1 and O2, which can be explained by the hybridization between the Cu 3d and the O 2p orbital, which enhances the magnetic moments of the anions.

**Table 1:** Lattice Parameters (*a*, *b*, and *c*), Lattice Angles (α, β, and γ), Atomic Bader Charges (*q*), and Atomic Magnetic Moments (*m*_s_) for the Bulk Phase of CuWO_4_

properties	this work	previous works
*A*	4.69	4.69^47^
*B*	5.83	5.83^47^
*C*	4.90	4.88^47^
Α	91.69	91.64^47^
Β	92.22	92.41^47^
Γ	83.89	82.91^47^
*q*_0_(Cu) (e^–^/atom)	1.24	1.17^48,49^
*q*(W) (e^–^/atom)	2.90	2.90^48,49^
*q*(O) (e^–^/atom)	–1.04	–1.37^48,49^
*m*_s_(Cu) (μ_B_ atom)	0.78	0.74^50^
*m*_s_(O) (μ_B_ atom)	0.03	0.068^50^

**Figure 2:**
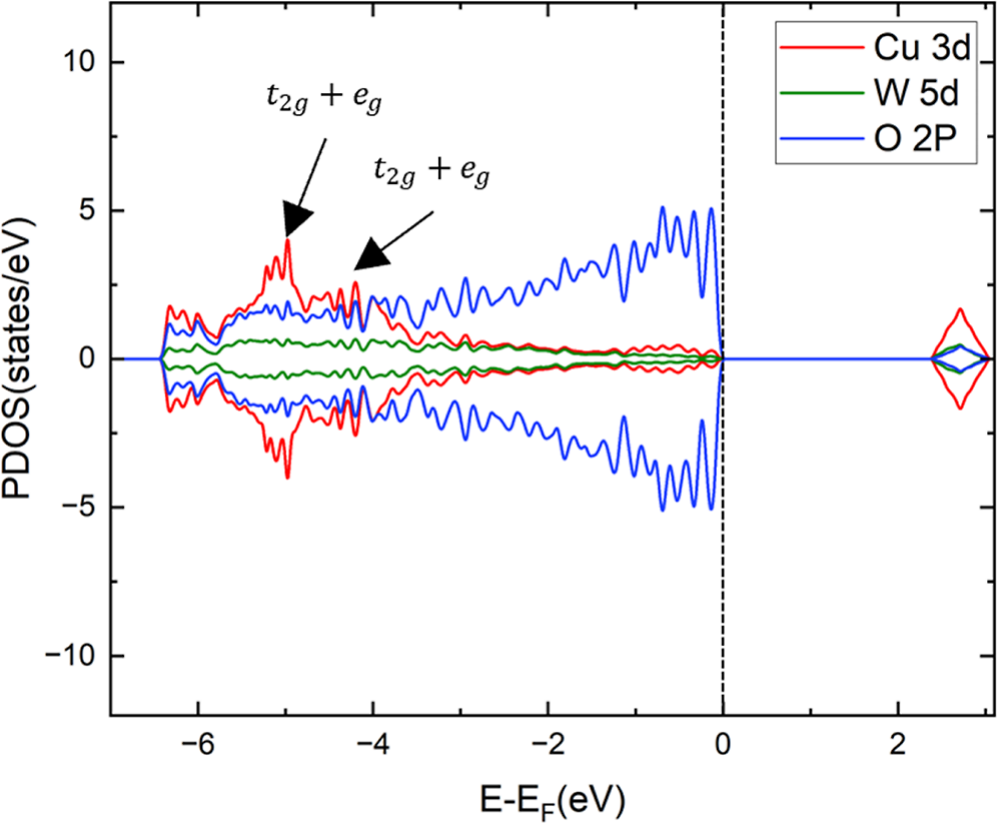
Projected DOS of the bulk phase of CuWO_4_, with the Fermi level indicated by a dashed line at 0 eV.

The density of states (DOS) of CuWO_4_ is shown in Figure 2. The valence band is mainly composed of O 2p states mixed with Cu 3d orbitals positioned between −6.5 and −4 eV. The conduction band, which lies between 2.3 and 3.1 eV, mainly comprises Cu 3d states mixed with the O 2p orbitals and W 5d levels. The on-site Coulomb repulsion separates the occupied levels from the empty Cu 3d-states, resulting in a bandgap of 2.3 eV, which agrees with previous studies.^29,30,50–52^

### 3.2 Pristine Surfaces of CuWO_4_

We used METADISE to obtain the nonpolar, symmetric, and stoichiometric terminations of the pristine CuWO_4_ surfaces. Based on the dipole model classification, we divided the surfaces into two groups, that is, the Tasker type 1 (101) and Tasker type 2 (001) and (010) facets, and the Tasker type 3 (011), (100), (110), and (111) facets. We have calculated the surface energy, atomic Bader charge, magnetic moment, and work function for each slab and tabulated the results in Table 2.

**Table 2:** Surface Energy (γ_r_), Atomic Bader Charge (*q*), Magnetic Moment (*m*_s_), Work Function (Φ), and Relaxation (*R*) of the Two Possible Nonpolar Symmetric and Stoichiometric Terminations of Each Pristine Low-Miller Index Surface of CuWO_4_

			*q* (e^–^/atom)			*m*_s_ (μ_B_/atom)			
surface	termination	γ_r_ (eV/Å^2^)	Cu	W	O	Cu	W	Φ (eV)	*R* (%)
001	(O_2_)_1_	0.073	1.25	2.28	–1.02	0.81	0.01	6.11	38
	(O_2_)_2_	0.065	1.23	2.81	–1.01	–0.77	0.01	6.80	64
010	CuO_2_	0.036	1.25	2.89	–1.03	–0.80	–0.01	6.95	33
	WO_2_	0.044	1.24	2.80	–1.01	0.75	0.01	8.27	66
011	WO_2_	0.098	1.20	2.78	–0.99	0.72	0.003	8.77	64
	CuO_2_	0.062	1.21	2.87	–1.02	0.77	0.02	6.51	41
100	Cu	0.068	1.17	2.88	–1.02	0.76	0.001	6.52	42
	W	0.195	1.20	2.53	–0.93	–0.69	0.58	6.80	39
101	(CuWO_4_)_1_	0.064	1.18	2.80	–1.00	0.57	–0.02	8.94	72
	(CuWO_4_)_2_	0.102	1.20	2.80	–1.00	–0.63	0.01	6.41	42
110	(CuWO_4_)_1_	0.069	1.21	2.81	–1.01	–0.65	0.01	8.19	64
	(CuWO_4_)_2_	0.058	1.25	2.84	–1.02	–0.79	0.001	6.56	54
111	Cu_2_W_2_O_2_	0.108	1.14	2.76	–0.97	–0.39	0.01	5.82	58
	O_6_	0.069	1.22	2.80	–1.00	–0.81	0.01	4.58	61

**Figure 3:**
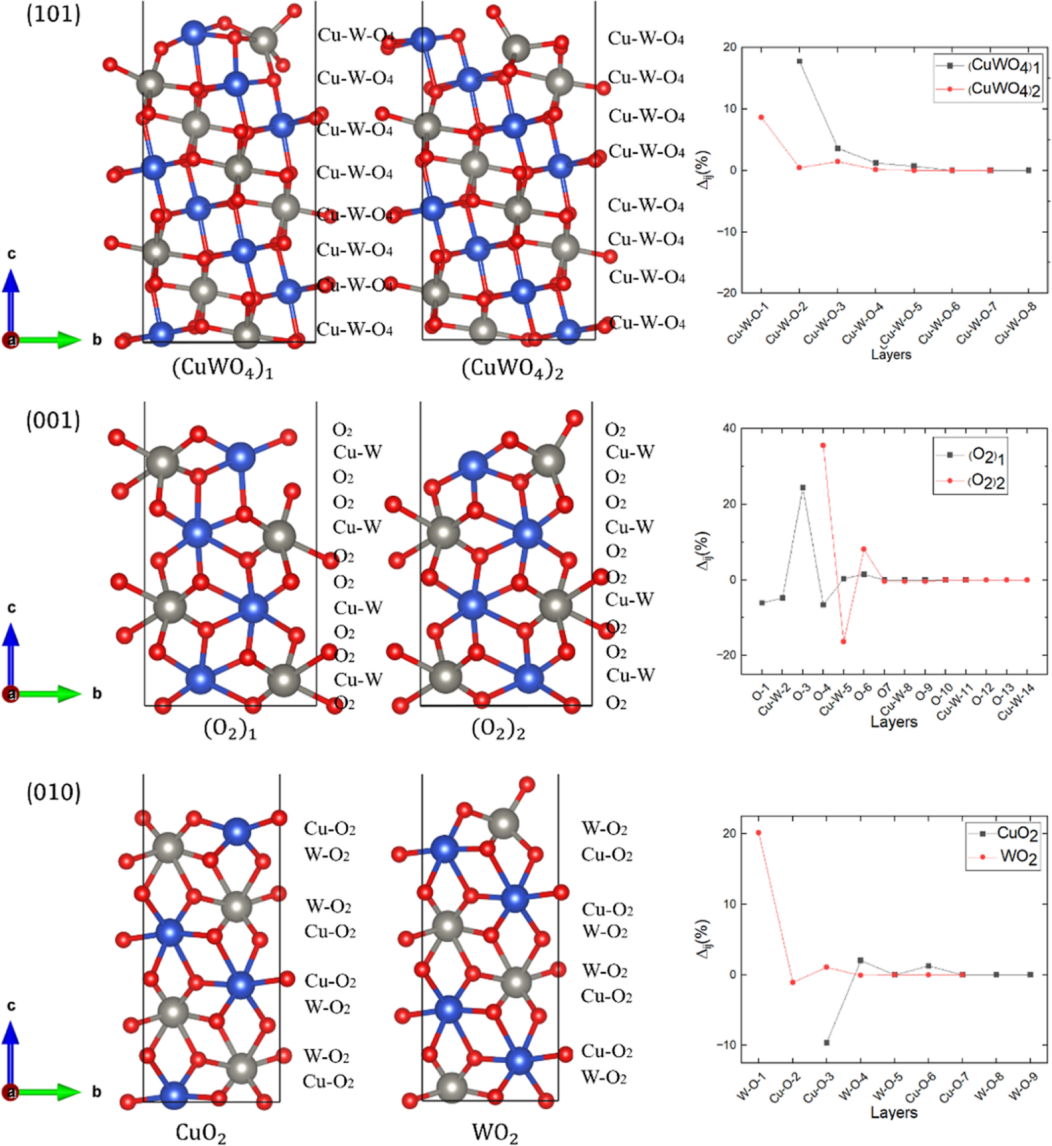
(Left and middle panels) Side views and (right panels) relaxation of the interplanar distances of the two possible nonpolar, symmetric, and stoichiometric terminations for the Tasker type 1 (101) and Tasker type 2 (001) and (010) surfaces. Cu, W, and O atoms are represented in blue, gray, and red, respectively. The stacking sequence of each termination is shown alongside a side view.

Figure 3 displays the side views alongside the stacking sequence and relaxation of the interplanar distances for the Tasker type 1 and 2 surfaces, that is, the (101), (001), and (010) surfaces.

The (101) surface, which is Tasker type 1, has two nonpolar symmetric terminations (CuWO_4_)_1_ and (CuWO_4_)_2_, which are constructed by (CuWO_4_) layers with slightly different structures, as depicted in Figure 3. Cu has two dangling bonds, and W has one dangling bond in termination (CuWO_4_)_1_, whereas W has two dangling bonds, and Cu has one dangling bond in termination (CuWO_4_)_2_. After relaxation, we found that only the topmost Cu–W–O-1 layer is pulled 8.64% inward to maintain the octahedral coordination in termination (CuWO_4_)_2_. For termination (CuWO_4_)_1_, the topmost Cu–W–O-2 layer moves 17.77% outward to maintain the octahedral coordination. The subsurface Cu–W–O-3 and Cu–W–O-4 planes are pulled 3.58 and 1.25%, respectively, toward the surface. The calculated atomic Bader charges and magnetic moments for the two nonpolar terminations of the (101) surface are listed in Table 2. Our result suggests that the changes in the atomic Bader charges of the exposed atoms in both terminations are less than 0.01 e^–^/atom after relaxation compared to their bulk counterpart, indicating that the ionic characteristic of the material does not change at the (101) surface. For termination (CuWO_4_)_1_, the magnetic moments calculated for Cu and W were 0.57 and −0.02 μ_B_/atom, respectively. For termination (CuWO_4_)_2_, we found that the magnetic moment for Cu is only 0.06 μ_B_/atom larger than in termination (CuWO_4_)_1_ but aligned in the opposite direction, whereas W remains almost nonmagnetic. Although the work function obtained for termination (CuWO_4_)_2_ is 2.51 eV smaller than that for termination (CuWO_4_)_1_, the former is ignored in the following analysis due to its larger surface energy, which indicates lower stability.

The (001) surface, which is Tasker type 2, has two nonpolar and symmetric terminations (O_2_)_1_ and (O_2_)_2_ that are composed of alternating Cu–W and O layers with both undercoordinated Cu and W having a single dangling bond each, see Figure 3. After relaxation, termination (O_2_)_1_ shows a 6.07% inward movement of the topmost negatively charged O-1 layer, which experiences Coulomb attraction to the positive subsurface Cu–W-2 layer. We found that the following Cu–W-2 and O-3 layers move 4.8% inward and 24.4% outward, respectively, due to the Coulomb attraction between them. The O-4 layer is pulled 6.6% inward because of the lower electrostatic attraction from the Cu–W-1 layer. For termination (O_2_)_2_, the topmost O-4 layer is pushed outward by 35.6% to maintain the octahedral coordination. The following Cu–W-5 and O-6 layers move 16.3% inward and 8.1% outward, respectively, due to the Coulomb attraction between these layers. The electronic properties of the (001) surface are summarized in Table 2. After relaxation of the (O_2_)_1_ termination, we found that the atomic Bader charges change by less than 0.1 e^–^/atom for the undercoordinated Cu, W, and O, whereas the magnetic moments for the exposed Cu and W are 0.81 and 0.01 μ_B_/atom, respectively. Our calculations suggest that the atomic Bader charges of Cu, W, and O do not change significantly after relaxation of termination (O_2_)_2_. We found that the magnetic moments for Cu and W sited in the topmost layer are −0.77 and 0.007 μ_B_/atom, respectively. Termination (O_2_)_1_ has a slightly lower work function (6.1 eV) compared to termination (O_2_)_2_ (6.8 eV), indicating that it requires less energy to remove the loosely held electron during photocatalysis. However, termination (O_2_)_1_ is not considered for further analysis due to its high relaxed surface energy, which indicates its lack of stability.

The (010) surface, which is a Tasker type 2, also has two terminations, namely, CuO_2_ and WO_2_, that are constructed from alternating cation–oxygen-mixed layers (CuO_2_) and (WO_2_), see Figure 3. Cu has two dangling bonds in termination CuO_2_, whereas W and Cu have one dangling bond each in termination WO_2_. In termination WO_2_, we found a large 20.18% outward relaxation and small 1% inward displacement for the topmost positively charged W–O-1 layer and the negatively charged Cu–O-2 subsurface plane, respectively, in order to maintain the bulk octahedral coordination. After relaxation, the topmost negatively charged Cu–O-3 layer in termination CuO_2_ undergoes a 9.63% inward relaxation to maintain its bulk octahedral coordination. The following positively charged W–O-4 layer is pushed by 2.02% toward the surface due to the Coulomb attraction with the Cu–O-3 layer. The calculated atomic Bader charges and magnetic moments of the two possible nonpolar terminations of the (010) surface are listed in Table 2. The atomic Bader charges for the exposed atoms in both terminations remain very similar compared to those in the bulk, suggesting that the ionic characteristic of CuWO_4_ does not change at the (010) surface. We found that the magnetic moments for Cu, although oriented in opposite directions, are slightly larger in termination CuO_2_ than in termination WO_2_, whereas the magnetic moment for W is essentially the same. The calculated work functions for terminations CuO_2_ and WO_2_ are 6.945 and 8.266 eV, respectively, which indicate that the former is more reactive than the latter and is able to provide more easily the electrons required for the photocatalytic processes. Thus, termination WO_2_ is not considered for further analysis due to its large surface energy and work function.

**Figure 4:**
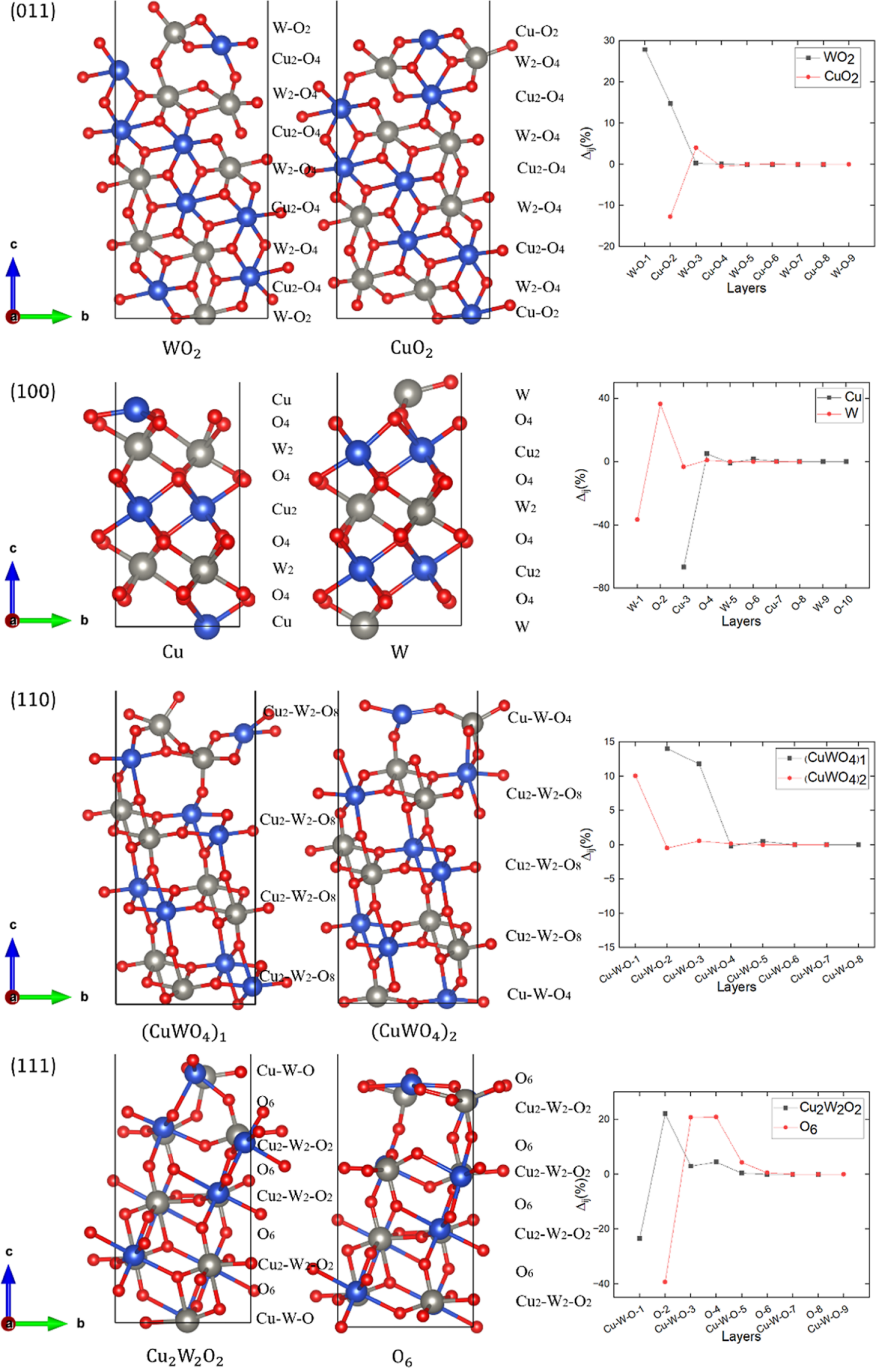
(Left and middle panels) Side views and (right panels) relaxation of the interplanar distances of the two possible reconstructed, nonpolar, symmetric, and stoichiometric terminations for the Tasker type 3 (011), (100), (110), and (111) surfaces. Cu, W, and O atoms are represented in blue, gray, and red, respectively. The stacking sequence of each termination is shown alongside the side view.

The side views and the relaxation of the interplanar distances of the reconstructed (011), (100), (110), and (111) slabs, which are Tasker type 3 surfaces, are shown in Figure 4.

The (011) surface, which is a Tasker type 3, has two nonpolar symmetric terminations W_2_O_2_ and Cu_2_O_2_, which are reconstructed by moving half of the O atoms from the topmost layer to the bottom of the slab, as depicted in Figure 4. Two terminations are possible due to the alternating stacking of the (W_2_O_4_) and (Cu_2_O_4_) layers along the [011] direction, characterized by exposed Cu and W atoms with three dangling bonds. We found that the topmost positively charged W–O-1 layer of termination W_2_O_2_ moves 27.90% outward during relaxation to keep its octahedral coordination. The subsurface negatively charged Cu–O-2 layer moves by 14.80% toward the surface due to the small Coulomb attraction from the W–O-1 layer. In termination Cu_2_O_2_, the topmost negatively charged Cu–O-2 layer undergoes 12.78% inward relaxation as its atoms with dangling bonds aim to increase their coordination number. The following positively charged W–O-3 layer moves by 4.01% toward the surface due to the Coulomb attraction from the Cu–O-2 layer above. The atomic Bader charges and magnetic moments for the two nonpolar terminations of the (011) surface are listed in Table 2. Our results suggest that the ionic characteristic does not change noticeably for the (011) surface since the differences in the atomic Bader charges between the exposed atoms on the topmost layer and the bulk are negligible. We found that the magnetic moments of Cu and W in termination Cu_2_O_2_ are slightly larger than in termination W_2_O_2_. The work function of termination Cu_2_O_2_ is 6.51 eV, which is 2.26 eV smaller than that in termination W_2_O_2_. Thus, termination Cu_2_O_2_ is selected for the following analyses owing to its small surface energy and work function.

The (100) surface, which is also a reconstructed Tasker type 3, has two nonpolar terminations, that is, Cu and W that are terminated by cation layers with three dangling bonds alternating with the O layers, see Figure 4. For termination W, the topmost positively charged W-1 layer is pulled inward by 36.60%, whereas the following negatively charged O-2 layer moves by 36.66% toward the surface in order to maintain the octahedral coordination of the atoms in the W-1 layer above. The following positively charged Cu-3 layer moves inward by 3.41% due to a modest Coulomb attraction from the O-4 layer below. In termination Cu, the topmost positively charged Cu-3 layer suffers a 66.62% relaxation inward owing to the Coulomb attraction from the subsurface O-4 plane, which moves outward by only 5.06%. The atomic Bader charges and magnetic moments for the two nonpolar terminations of the (100) surface are listed in Table 2. The atomic Bader charges of the exposed atoms on both terminations change by less than 0.01 e^–^/atom during relaxation, suggesting that the ionic characteristic does not change for the (100) surface with respect to the bulk. In termination Cu, the magnetic moment of Cu is 0.76 μ_B_/atom, which is larger than the magnetic moment of 0.69 μ_B_/atom in termination W. However, the magnetic moment of W in termination Cu is 0.001 μ_B_/atom, indicating it to be nonmagnetic and smaller than in termination W. The work function of termination Cu is 6.52 eV, which is 0.41 eV smaller than that for termination W, suggesting that the former surface is slightly more reactive than the latter. Thus, termination W is not considered further in the following sections due to its larger surface energy and work function.

The (110) surface, which is a Tasker type 3 surface, has two nonpolar terminations (CuWO_4_)_1_ and (CuWO_4_)_2_, which are terminated by alternating (Cu_2_W_2_O_8_) and (CuWO_4_) layers, as shown in Figure 4. Our models indicate that the exposed Cu and W atoms had two dangling bonds each. In termination (CuWO_4_)_2_, the topmost Cu–W–O-1 layer moves outward by 10.06% to keep the octahedral coordination of Cu and W, whereas the other planes changed their interplanar distances by less than 1%. As for termination (CuWO_4_)_1_, the topmost Cu–W–O-2 layer moves by 14.01% toward the surface to keep the octahedral coordination of the cations. The subsurface Cu–W–O3 layer is pulled outward by 11.80% due to the Coulomb attraction from the Cu–W–O-2 layer above. The atomic Bader charges and magnetic moments of the two nonpolar terminations of the (110) surface are listed in Table 2. Our results show that the atomic Bader charges of the two terminations change by less than 0.1 e^–^/atom upon relaxation compared to the bulk, indicating that the ionic characteristic does not change noticeably on the (110) surface. Our results suggest that the magnetic moment of Cu in termination (CuWO_4_)_2_, despite its orientation, is smaller than that in termination (CuWO_4_)_1_, which is more magnetic. However, the magnetic moments of W, although oriented in opposite directions, are essentially the same. The work functions of terminations (CuWO_4_)_1_ and (CuWO_4_)_2_ are 8.19 and 6.56 eV, respectively. Hence, termination (CuWO_4_)_2_ is not considered for further analysis due to its large surface energy and work function.

The (111) surface has two nonpolar terminations Cu_2_W_2_O_2_ and O_6_, which are terminated by the (Cu_2_W_2_O_2_) layer of mixed cations and the O layer, with three dangling bonds of exposed Cu and W in the topmost layers of both terminations, see Figure 4. In termination Cu_2_W_2_O_2_, the negatively charged O-2 layer moves outward by 22.14% to allow the cations of the Cu–W–O-1 layer to maintain the octahedral coordination of the bulk, which in turn are pulled in by 23.41%. The positively charged subsurface Cu–W–O-3 layer moves by 2.99% due to the lower Coulomb attraction from the O-2 layer. The negatively charged O-4 layer is pushed outward by 4.46% due to the small Coulomb attraction from the Cu–W–O-3 layer above. In termination O_6_, we found that the topmost negatively charged O-2 layer moves slightly toward the bulk. However, the positively charged subsurface Cu–W–O-3 layer undergoes a 20.80% outward relaxation to maintain the octahedral coordination of the cations in the bulk by becoming closer to the topmost O-2 layer, which is pulled inward by 39.38%. Due to the large Coulomb attraction from the Cu–W–O-3 layer, the O-4 moves by 20.98% toward the surface. The following positively charged Cu–W–O-5 layer is then pulled 4.28% outward due to the Coulomb attraction from the O-4 layer above. The atomic Bader charges and magnetic moments of both nonpolar terminations are listed in Table 2. Negligible changes in the atomic Bader charges of both terminations are observed after relaxation, suggesting that the ionic characteristic does not change for the (111) surface. Regardless of the direction of orientation, the magnetic moment of Cu in termination O_6_ is larger than that in termination Cu_2_W_2_O_2_. However, the magnetic moments of W in the two terminations are the same and negligible. The work functions are 5.82 and 4.58 eV in terminations Cu_2_W_2_O_2_ and O_6_, respectively, which indicate that the anion-terminated surface is more reactive. Thus, termination O_6_ is selected for the following analyses because of the relatively smaller surface energy and work function.

### 3.3 Redox Properties of CuWO_4_

**Figure 5:**
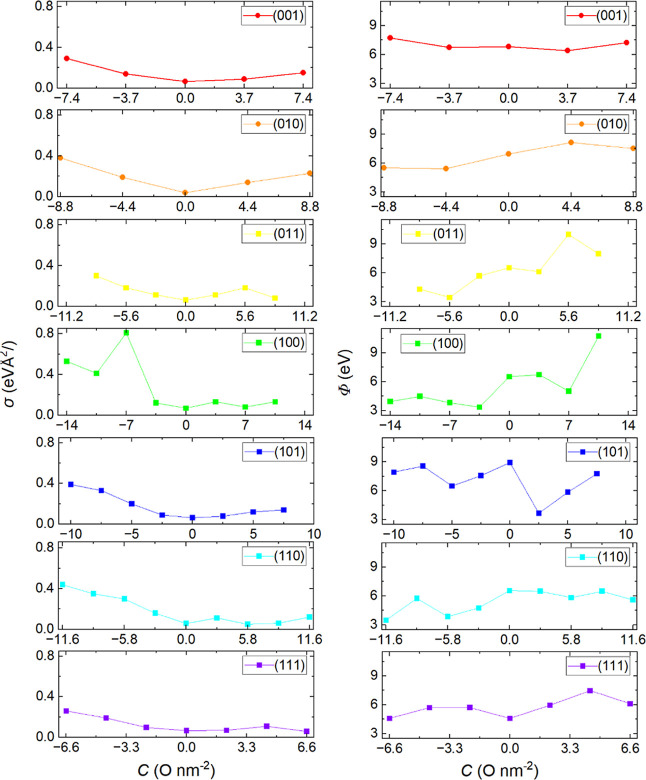
Surface free energy (σ) at 0 K (left) and work function (Φ) (right) for each coverage of O adatoms and vacancies for the low-Miller index surfaces of CuWO_4_. The coverages of the O adatoms and O vacancies of the nonpristine surfaces are noted on the axes in the figures by positive and negative values, respectively.

Here, we analyze the redox properties of the thermodynamically most stable terminations of each pristine surface. Oxygen atoms were sequentially added to the exposed cations with dangling bonds on each surface to simulate different degrees of oxidation, with the maximum number of oxygen atoms added corresponding to the number of dangling bonds present on the surface. To simulate the partial reduction, oxygen atoms were selectively removed from only the topmost layer of each surface. The entire structure was fully relaxed for each degree of partial oxidation and reduction, and the atomic configuration with the lowest surface free energy was chosen to model the surface with the next coverage of the O adatoms or the O vacancies. The electronic properties, including atomic Bader charges (*q*) and magnetic moments (*m*_s_) for each oxygen coverage (*C*) of the low-Miller index surfaces are presented in Table 3, whereas the surface free energy (σ) and work function (Φ) are depicted in Figure 5. The surface free energy of the partially oxidized (σ_oxi-0K_) and partially reduced (σ_red-0K_) surfaces at 0 K are defined as
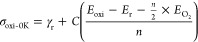
6
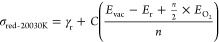
7where  is the coverage of oxygen vacancies (when negative) or the coverage of oxygen adatoms (when positive) and *n* represents the number of oxygen vacancies or oxygen adatoms. *E*_oxi_ and *E*_vac_ are the energies of the partially oxidized and partially reduced surface slabs, whereas *E*_O_2__ is the energy of the oxygen molecule calculated by DFT.

**Table 3:** Atomic Bader Charges (*q*) and Magnetic Moments (*m*_s_) for Each Oxygen Coverage (*C*) of the Low-Miller Index Surfaces of CuWO_4_

		*q* (e^–^/atom)			*m*_s_ (μ_B_/atom)	
surface	*C* (O nm^–2^)	Cu	W	O	Cu	W
(001)	7.4	1.18	2.81	–0.89	0.71	0.02
	3.7	1.23	2.82	–0.95	0.78	0.01
	–3.7	0.96	2.76	–0.99	0.00	0.00
	–7.4	0.83	2.63	–0.99	–0.01	–0.66
(010)	8.8	1.25	2.89	–0.92	0.67	0.01
	4.4	1.24	2.89	–0.97	0.40	0.02
	–4.4	0.99	2.81	–1.02	0.00	0.00
	–8.8	0.89	2.64	–1.01	0.01	–2.37
(011)	8.4	1.24	2.87	–0.94	–0.77	0.00
	5.6	1.22	2.87	–0.96	–0.76	–0.09
	2.8	1.24	2.87	–1.00	–0.71	–0.04
	–2.8	1.11	2.85	–1.02	0.00	–0.01
	–5.6	0.98	2.81	–1.01	0.00	0.00
	–8.4	0.93	2.67	–0.99	0.06	0.00
(100)	10.5	1.17	2.87	–0.85	–0.62	–0.01
	7.0	1.12	2.87	–0.89	–0.74	0.00
	3.5	1.14	2.87	–0.94	–0.71	0.00
	–3.5	0.95	2.81	–1.00	0.00	–0.32
	–7.0	0.84	2.68	–1.01	0.00	–0.05
	–10.5	0.74	2.53	–1.01	0.01	–0.01
	–14.0	0.63	2.41	–1.01	0.00	0.06
(101)	7.5	1.23	2.80	–0.92	0.71	0.02
	5.0	1.18	2.79	–0.93	–0.70	0.03
	2.5	1.20	2.81	–0.97	–0.67	0.00
	–2.5	1.12	2.77	–1.00	–0.05	–0.04
	–5.0	0.98	2.70	–0.98	–0.07	–0.12
	–7.5	0.98	2.63	–1.00	0.92	–1.76
	–10.0	0.91	2.58	–1.00	0.00	–0.28
(110)	11.6	1.25	2.85	–0.91	0.75	0.07
	8.7	1.24	2.84	–0.93	0.69	0.00
	5.8	1.25	2.84	–0.96	–0.72	–0.02
	2.9	1.21	2.83	–0.98	2.73	0.05
	–2.9	1.17	2.76	–1.01	–0.10	–0.23
	–5.8	1.10	2.71	–1.02	–0.03	–0.68
	–8.7	0.96	2.71	–1.01	–0.05	0.31
	–11.6	0.92	2.62	–1.01	–0.08	–0.24
(111)	6.6	1.22	2.80	–0.92	–0.81	0.00
	4.4	1.23	2.79	–0.95	–0.73	0.03
	2.2	1.22	2.80	–0.97	0.80	0.74
	–2.2	1.07	2.79	–1.00	0.00	0.00
	–4.4	1.01	2.72	–1.00	0.00	0.01
	–6.6	0.92	2.63	–0.98	–0.04	–0.21

We found that the atomic Bader charges exhibit slight fluctuations within a range of 0.1 eV during oxidation, which closely corresponds to the extent of structural changes after relaxation. However, during reduction, the atomic Bader charges of the cations decrease in each surface as the number of O vacancies is created, resulting in fewer electron donors. Our calculations show that the magnetic moments of Cu and W undergo minor changes within 0.1 μ_B_/atom upon surface oxidation for most coverages of O adatoms. However, during reduction, the magnetic moment of Cu vanishes for almost all coverages of the O vacancies. In contrast, W becomes magnetic on the partially reduced (001), (010), (101), (110), and (111) surfaces. The substantial changes in the magnetic moments of the cations can be attributed to significant surface distortions that occur during reduction.

Figure 5 shows the surface free energy at 0 K and the work function for each coverage of O adatoms and vacancies in the low-Miller index surfaces. We found that the pristine surfaces exhibit the lowest surface free energies, whereas the partially oxidized surfaces generally have smaller surface free energies than the reduced systems, indicating that the former are thermodynamically more stable than the latter.

Our estimated surface free energy increases by approximately 0.3 eV/^2^ in both the partially oxidized and reduced (001) surfaces. We found the lowest work function of 6.39 eV for the surface with a coverage of O adatoms of 3.7 O nm^–2^. Upon partial oxidation and reduction, the surface free energies of the (010) surface increase by approximately 0.4 eV/Å^2^. The oxidized surface has a work function of more than 7.5 eV, indicating low photocatalytic reactivity. The partially reduced surface with a coverage of O vacancies of 4.4 O nm^–2^ possesses the lowest work function of 5.41 eV. In the (011) facet, the surface free energy remains below 0.4 eV/Å^2^ for coverages of O adatoms ranging from 0 to 8.4 O nm^–2^ and for coverages of O vacancies ranging from 8.4 to 0 O nm^–2^. The work function decreases with coverage of O vacancies in the partially reduced surfaces, with the lowest work function of 3.43 eV obtained for a vacancy coverage of 5.6 O nm^–2^. In the (100) surface, a significant increase in surface free energy is observed when two or more O atoms are removed during reduction. The surface free energy of the reduced surface with a vacancy coverage of 7.0 nm^–2^ is calculated to be 0.81 eV/^2^, indicating a substantial structural change caused by the movement of Cu in the topmost layer. The lowest work function of 3.35 eV is achieved when the partially reduced (100) surface has a coverage of the O vacancies of 3.7 O nm^–2^. In the (101) facet, the surface free energy remains below 0.4 eV/^2^ for any coverage of O adatoms or O vacancies, and the lowest work function of 3.67 eV is attained for the partially oxidized surface with a coverage of O adatoms of 2.5 O nm^–2^. In the (110) surface, the lowest work function of 3.47 eV is obtained for the partially reduced surface with a coverage of the O vacancies of 11.6 O nm^–2^, despite having the largest surface free energy of 0.44 eV/Å^2^. Finally, in the (111) plane, the surface free energy remains below 0.26 eV/^2^ for all the coverages of O adatoms and vacancies explored in this work, with the pristine surface showing the lowest surface free energy of 4.58 eV.

### 3.4 Surface Phase Diagrams

#### 3.4.1 Surface Phase Diagrams under O_2_ Conditions

The redox processes of the CuWO_4_ surfaces under the conditions of O_2_ can be described by the following chemical reactions
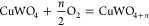
8
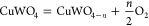
9

Based on these reactions, and assuming an ideal gas behavior, the surface free energies of the partially oxidized (σ_oxi_(*T*,*p*)) and partially reduced (σ_red_(*T*,*P*)) CuWO_4_ surfaces are calculated using the following equations
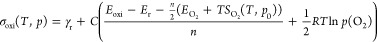
10
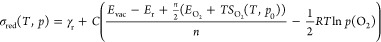
11where ln *p*(O_2_) is the logarithm of the partial pressure of O_2_, *S*_O_2__(*T*,*p*_0_) is the entropy of the oxygen molecule in the standard state, *T* is the temperature, and *R* represents the ideal gas constant.

**Figure 6:**
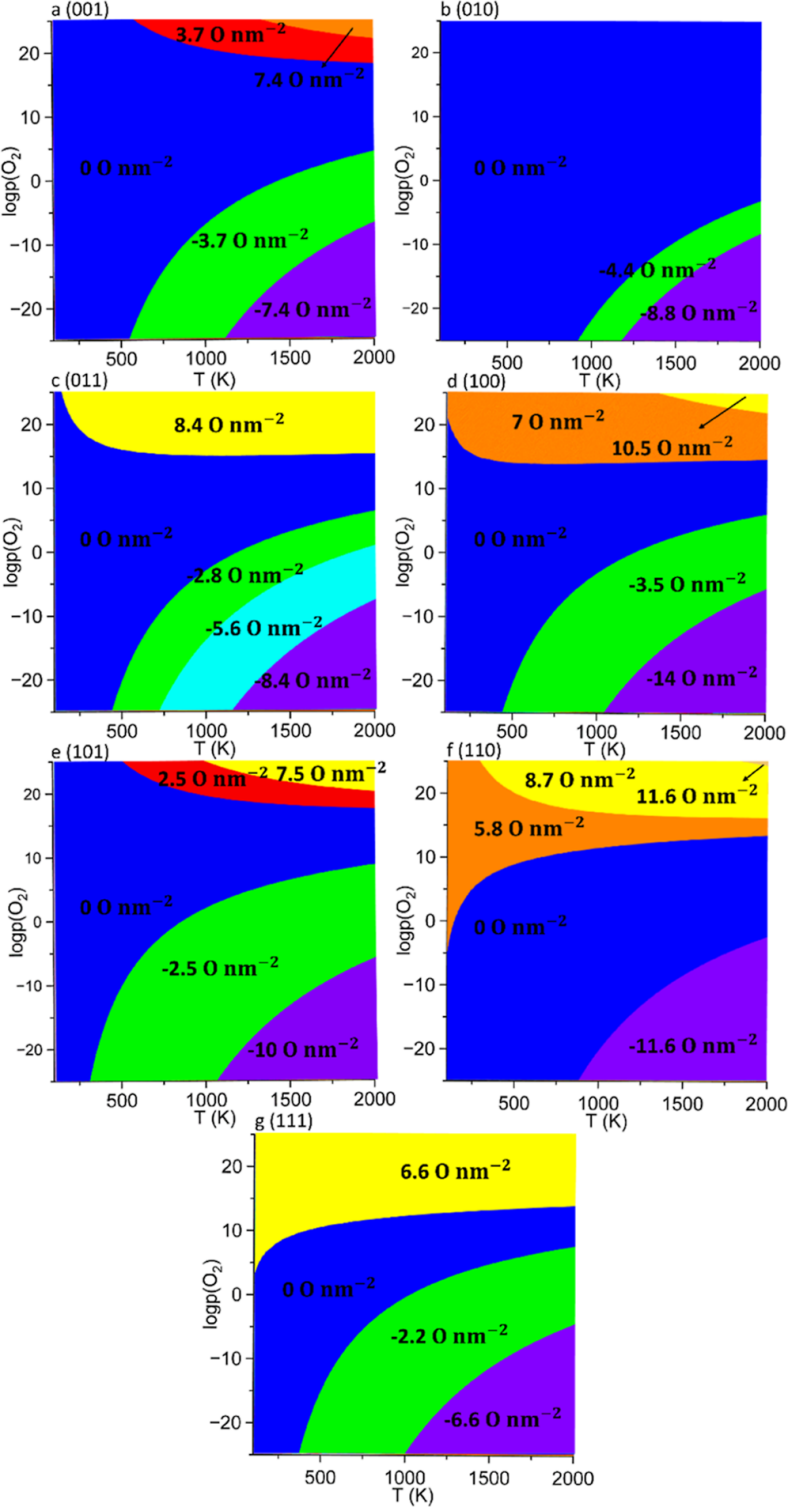
Surface phase diagrams for the low-Miller index surfaces of CuWO_4_ as a function of the logarithm of the partial pressure (*p*) of oxygen and temperature (*T*). The coverages (*C*) of the O adatoms and O vacancies for the nonpristine surfaces are noted in the figures with positive and negative values, respectively.

The surface face diagrams under oxygen conditions are constructed for each surface based on the atomic configurations with the lowest surface free energy calculated using eqs 10 and 11. The 2D surface-phase diagrams represent a bidimensional projection onto the plane formed by the temperature and log *p*(O_2_) of the 3D surface phase diagrams, see Figure S1 for an example. To provide a more intuitive and convenient representation, 2D projections of the surface phase diagrams are plotted as a function of the temperature and partial pressure of oxygen to illustrate the redox properties of each surface, as shown in Figure 6. The curves between the intersecting energy surfaces represent the conditions required to modify the extent of partial reduction or oxidation of each surface.

The (001) surface remains pristine when the temperature is below 500 K. As the temperature increases, the surface undergoes oxidation or reduction depending on the partial pressure of O_2_. When log *p*(O_2_) is larger than 17 and the temperature is above 600 K, the surface begins to oxidize, with a coverage of O adatoms of 3.7 nm^–2^, as shown in the red region in Figure 6a, indicating the adsorption of a single oxygen atom at our computational slab. As the temperature and log *p*(O_2_) continue to increase to 1400 K and 22, respectively, the coverage of the O adatoms reaches 7.4 O nm^–2^, corresponding to the adsorption of two oxygen atoms in our surface model. The surface undergoes reduction when the temperature is above 600 K, and log *p*(O_2_) is below 5 as shown in the green region in Figure 6a. The coverage of O vacancies is 3.7 O nm^–2^ when one oxygen atom is removed from the computational slab. As log *p*(O_2_) decreases below −7 and the temperature is above 1200 K, the coverage of O vacancies becomes 7.4 O nm^–2^ due to the loss of two oxygen atoms from the simulation cell. For all other conditions, the (001) surface remains pristine, as shown in the blue region in Figure 6a.

The (010) surface was found to remain pristine when the temperature is below 900 K and log *p*(O_2_) is above −4, as depicted by the blue region shown in Figure 6b. As the temperature increases and log *p*(O_2_) decreases to 1000 K and −4, respectively, the surface undergoes reduction with a coverage of O vacancies of 4.4 nm^–2^, as shown in the green region displayed in Figure 6b. When the temperature exceeds 1200 K and log *p*(O_2_) is below −9, the coverage of O vacancies increases to 8.8 O nm^–2^, corresponding to the loss of two oxygen atoms from our computational cell. Our simulations suggest that the (010) surface cannot be oxidized under the range of conditions considered in this study since two oxygen atoms are shielding the exposed Cu^2+^ cations, which are therefore inaccessible for the adsorption of environmental oxygen, as displayed in Figure 6b. Tests carried out under extreme conditions reveal that the (010) surface becomes partially oxidized when log *p*(O_2_) is larger than 30, as shown in Figure S2.

We found that the (011) surface always remains pristine when the temperature is lower than 600 K and only oxidizes with a coverage of adatoms of 2.8 O nm^–2^ when the temperature and log *p*(O_2_) are above 1400 K and 22, respectively, as displayed in the red region of Figure 6c. The (011) surface undergoes reduction with a coverage of O vacancies of 5.6 nm^–2^ when the temperature is above 600 K and log *p*(O_2_) is lower than 3, as depicted in the cyan-colored region in Figure 6c. The surface becomes fully reduced with a coverage of O vacancies of 8.4 O nm^–2^ when the temperature is above 1200 K and log *p*(O_2_) is under −8, indicating the loss of three oxygen atoms from our computational slab, as illustrated in the purple region of Figure 6c.

Our calculations suggest that the (100) surface can be oxidized by adsorbing two oxygen atoms at any temperature from 100 to 2000 to 2000 K when log *p*(O_2_) is above 14, resulting in a partially oxidized surface with a coverage of O adatoms of 7 O nm^–2^, as shown in the orange region of Figure 6d. We did not find evidence of a degree of oxidation equivalent to three oxygen adatoms, corresponding to a coverage of O adatoms of 10.5 O nm^–2^, since the oxygen atoms tend to form the oxygen dimer rather than adsorb onto the Cu^2+^ cation. The (100) surface starts reducing with a coverage of the number of O vacancies of 3.5 O nm^–2^ at 500 K when log *p*(O_2_) decreases below 6, corresponding to losing one oxygen atom from our computational model, as represented in the green region of Figure 6d. The surface loses a further three oxygen atoms when the temperature is above 1100 K and log *p*(O_2_) is below −6, respectively, resulting in a fully reduced surface with a coverage of O vacancies of 14 nm^–2^, as depicted in the purple region of Figure 6d.

The (101) surface stays pristine when the temperature is under 300 K. Increasing the temperature above 300 K reduces the surface to a coverage of O vacancies of 2.5 nm^–2^ when log *p*(O_2_) is below 8, as shown in the green region of Figure 6e. Increasing the temperature above 1100 K and decreasing log *p*(O_2_) below −6 fully reduce the (101) surface to a coverage of the O vacancies of 10 nm^–2^, as depicted in the purple region of Figure 6e. We found that the (101) surface starts to oxidize with a coverage of O adatoms of 2.5 O nm^–2^ when the temperature and log *p*(O_2_) increase above 600 K and 18, respectively. Increasing the temperature and log *p*(O_2_) above 1000 and 21 K results in a larger coverage of adatoms computed at 7.5 nm^–2^, corresponding to the adsorption of three oxygen adatoms at our computational slab, as represented in the yellow region of Figure 6e.

For the (110) surface, our results suggest that the partial oxidation with a coverage of O adatoms of 5.8 nm^–2^ begins at any temperature between 100 and 2000 K when log *p*(O_2_) is above 14, as shown in the orange region of Figure 6f. With an increase in temperature and log *p*(O_2_) to 300 K and 17, respectively, the (110) surface undergoes partial oxidization to a coverage of O adatoms of 8.7 nm^–2^, corresponding to the adsorption of three oxygen atoms in our simulation cell. When further increasing the temperature and log *p*(O_2_) above 1900 K and 24, the equilibrium coverage of O adatoms increases to 11.6 O nm^–2^, resulting in the complete oxidation of the surface with the adsorption of four oxygen adatoms at the computational slab, as shown in the region colored in dark yellow in Figure 6f. The surface then becomes pristine as the temperature and log *p*(O_2_) decrease simultaneously. When the temperature and log *p*(O_2_) decrease below 900 K and −3, respectively, the (110) surface becomes fully reduced with a coverage of O vacancies of 11.6 nm^–2^, as shown in the violet region of Figure 6f.

We found that the (111) surface can be oxidized with a coverage of only 6.6 nm of O adatoms of 6.6 O nm^–2^ when log *p*(O_2_) is above 14, corresponding to the adsorption of three oxygen atoms in our simulation cell, as shown in the yellow region of Figure 6g. Decreasing log *p*(O_2_) to −5 stabilizes the pristine surface, as shown in the blue region of Figure 6g. Reduction of the surface to a coverage of O vacancies of 2.2 O nm^–2^ is favored when the temperature is above 400 K and log *p*(O_2_) is below 7, as depicted in the green region of Figure 6g. We found that when log *p*(O_2_) decreases below −5, the (111) surface becomes fully reduced with a coverage of the O vacancies of 6.6 nm^–2^, corresponding to the creation of three oxygen vacancies in our computational slab, as illustrated in the purple region of Figure 6g.

Our results suggest that among all low-Miller index surfaces, the pristine (010) surface is thermodynamically the most stable plane with the lowest surface energy of 0.03 eV/^2^, within the widest range of temperatures and partial pressure of O_2_, as shown in Figure 6. On the other hand, the pristine (111) surface, which requires the narrowest range of temperatures and partial pressure of O_2_, has the lowest work function of 4.58 eV and is the most chemically reactive system compared to the other low-Miller index surfaces, see Figure 6.

#### 3.4.2 Surface Phase Diagrams under H_2_O/H_2_ Conditions

From a practical point of view, the water splitting reaction is more convenient in the gas phase than in solution since the former has a lower Gibbs free energy (−0.212 to 0.331 eV) for the adsorption of the water molecule, it reduces the possibility of corrosion of the catalyst, and it has small plasmonic thermal and near-infrared photothermal effects.^53,54^ In view of these benefits, we decided to analyze the redox surface phase diagrams for each low-Miller index surface of CuWO_4_ under gas-phase conditions for the water splitting process.

The reaction between gas-phase water and hydrogen with the CuWO_4_ surfaces can be represented as follows
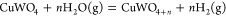
12
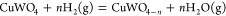
13

The surface free energies of CuWO_4_ under gas-phase water splitting conditions for the oxidized (σ_oxi_(*T*,*p*)) and reduced surfaces (σ_oxi_(*T*,*p*)) are calculated, assuming an ideal gas behavior, using the following equations
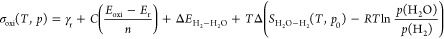
14
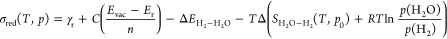
15where  is the logarithm of ratio of the partial pressure of H_2_O and H_2_, Δ*E*_H_2_–H_2_O_ is the energy difference between H_2_ and H_2_O from the DFT calculations, Δ(*S*_H_2_O–H_2__(*T*,*p*_0_)) represents the entropy difference between H_2_O and H_2_ at 1 bar based on thermodynamic tables,^55^ and R is the ideal gas constant. We have used the entropies of H_2_O and H_2_ calculated at different temperatures using statistical thermodynamics,^56^ which are in good agreement with the experimental values between 500 and 2000 K, see Figure S3.

**Figure 7:**
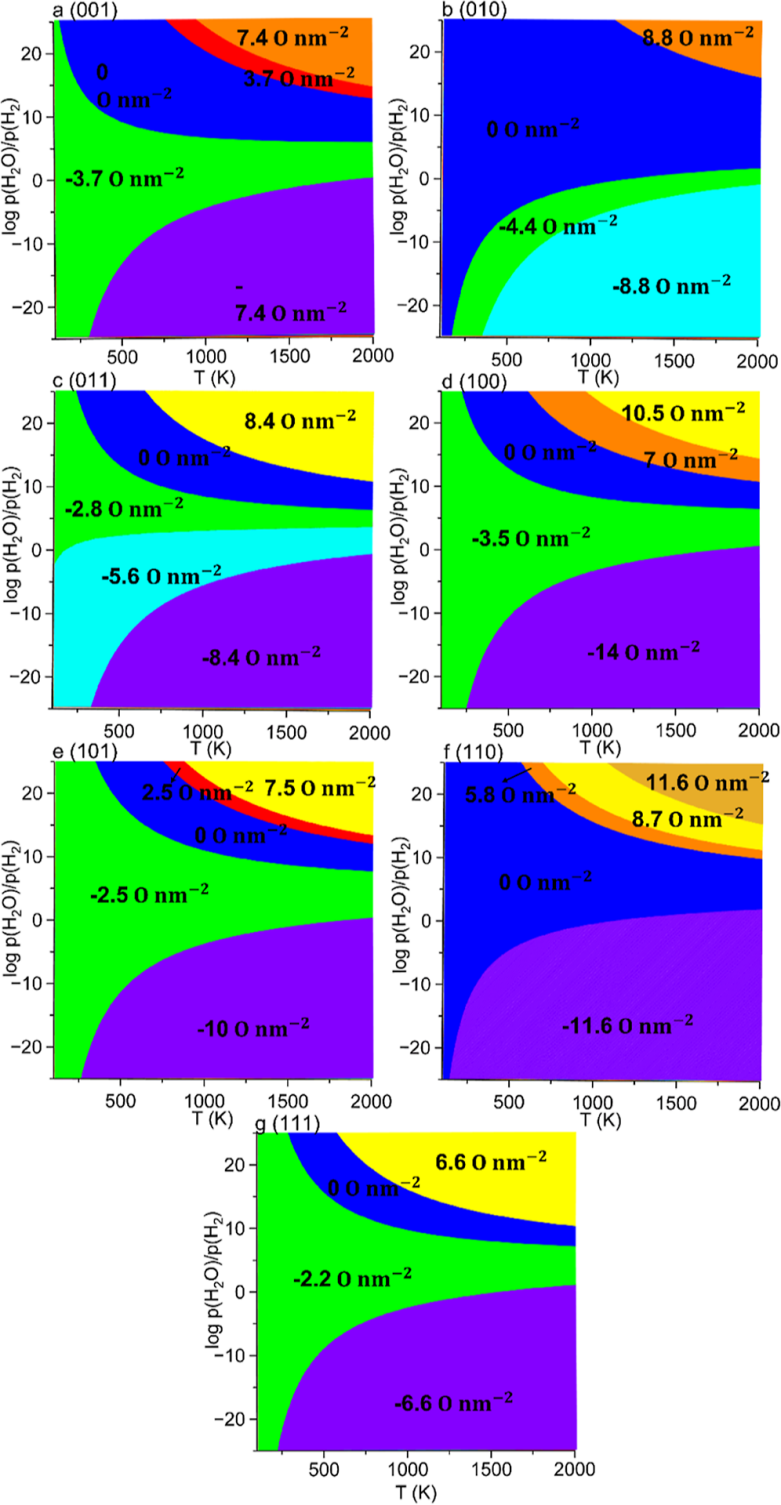
Surface phase diagrams for the low-Miller index surfaces of CuWO_4_ as a function of the logarithm of the ratio of the partial pressures (*p*) of H_2_O/H_2_ and temperature (*T*). The coverages (*C*) of the O adatoms and O vacancies for the nonpristine surfaces are noted in the figures with positive and negative values, respectively.

On the (001) surface, reduction begins at 100 K, resulting in a coverage of O vacancies of 3.7 O nm^–2^ at any temperature when  is between 1 and 5, as shown in Figure 7a. Further increasing the temperature and decreasing  to 298 K and 1, respectively, fully reduces the surface with a coverage of O vacancies of 7.4 O nm^–2^, shown as a purple region in Figure 7a. We found evidence that oxidation at a coverage of O adatoms of 3.7 O nm^–2^ occurs at a temperature and  above 750 K and 13, respectively. For a temperature and  above 1000 K and 15, respectively, the coverage of O adatoms increases to 7.4 O nm^–2^, corresponding to complete oxidation with the adsorption of two oxygen atoms in our simulation cell.

We found that the (010) surface undergoes partial reduction with a coverage of O vacancies of 4.4 O nm^–2^ when the temperature exceeds 200 K and  is below 10, as depicted in Figure 7b. As the temperature increases to 400 K and  decreases to −1, the surface becomes fully reduced, resulting in a coverage of O vacancies of 8.8 O nm^–2^, as shown in the cyan region in Figure 7b. Only one level of partial oxidation appears when the temperature and  are both above 1200 K and 17, respectively, resulting in a coverage of O adatoms of 8.8 O nm^–2^, equivalent to complete oxidation after adsorption of two oxygen atoms in our simulation cell, see the orange color region in Figure 7b.

A large region is predicted for the partially reduced (011) surface above 100 K, with a coverage of O vacancies of 2.8 O nm^–2^, as depicted in green in Figure 7c. The (011) surface loses another oxygen atom when  is below 2, corresponding to a coverage of O vacancies of 5.6 O nm^–2^. Increasing the temperature above 400 K and decreasing  below −1 increases the coverage of O vacancies to 8.4 O nm^–2^, indicating complete reduction after our simulation cell loses three oxygen atoms. Only one level of partial oxidation appears, at a coverage of O adatoms of 8.4 O nm^–2^, when the temperature and  are above 800 K and 11, as illustrated in the yellow region of Figure 7c.

The (100) surface undergoes partial reduction with a coverage of O vacancies of 3.5 O nm^–2^ at any temperature when  is between 1 and 6, represented as the green region in Figure 7d. Increasing the temperature above 250 K and decreasing  below 6 fully reduce the surface, resulting in a coverage of O vacancies of 14 O nm^–2^, corresponding to losing four oxygen atoms from our simulation cell. We found that the (100) surface starts to oxidize with a coverage of O adatoms of 7 O nm^–2^ when the temperature and  are above 700 K and 11, respectively. Further increasing the temperature to 1000 K and  to 15 oxidizes the surface fully with a coverage of O adatoms of 10.5 O nm^–2^ after the adsorption of three oxygen atoms in our simulation cell.

Our results suggest that the (101) surface is capable of becoming reduced with a coverage of O vacancies of 2.5 O nm^–2^ at any temperature between 100 and 2000 K when  is between 1 and 7, shown as a green region in Figure 7e. Increasing the temperature above 250 K and decreasing  below 1 fully reduces the (101) surface with a coverage of O vacancies of 10 O nm^–2^, corresponding to four oxygen vacancies in our simulation cell. We found that oxidation to a coverage of O adatoms of 2.5 O nm^–2^ occurs when the temperature and  are above 750 K and 13, respectively. Further increasing the temperature and  to above 900 K and 14 fully oxidizes the surface with a coverage of O adatoms of 7.5 O nm^–2^, corresponding to the adsorption of three oxygen atoms in our simulation cell, shown as a yellow region in Figure 7e.

Our calculations indicate that the (110) surface can only be reduced when the temperature is above 200 K and  is below 2, which corresponds to the complete reduction of the (110) surface, which loses four oxygen atoms, resulting in a coverage of O vacancies of 11.6 O nm^–2^, shown in the purple region of Figure 7f. We found that partial oxidation of the (110) surface, with a coverage of O adatoms of 5.8 O nm^–2^, occurs when the temperature and  are above 600 K and 10, respectively. Increasing the temperature and  above 700 K and 11, respectively, leads to partial oxidation of the (110) surface with a coverage of O adatoms of 8.7 O nm^–2^, corresponding to the adsorption of three atoms in our simulation cell. The surface becomes completely oxidized with a coverage of O adatoms of 11.6 O nm^–2^ when the temperature and  are larger than 1100 K and 15, respectively, shown as a dark yellow region in Figure 7f.

The (111) surface undergoes partial reduction to a coverage of O vacancies of 2.2 O nm^–2^ when  is between 1 and 7, as shown in the green region of Figure 7g. When  decreases below 1, the (111) surface is fully reduced with a coverage of O vacancies of 6.6 O nm^–2^, corresponding to three oxygen vacancies in our simulation cell. Our results show that oxidation, with a coverage of O adatoms of 6.6 O nm^–2^, becomes thermodynamically stable only when the temperature and  are above 600 K and 10, respectively, corresponding to the adsorption of three oxygen atoms in the simulation cell.

We found that the H_2_O/H_2_ environment facilitates reduction of the material since O_2_ is an oxidizing agent, whereas H_2_ has a strong reducing characteristic. With the exception of the (010) and (110) surfaces, the remaining facets become reduced at any temperature between 100 and 2000 K under relevant partial pressures of H_2_O and H_2_. In contrast, the surface phase diagram under O_2_ conditions shows that the (010) surface is oxidized when both the temperature and  exceed 1200 K and 17. However, our results suggest that it is difficult to oxidize the CuWO_4_ surface under H_2_O/H_2_ conditions unless a high partial pressure of H_2_O and elevated temperatures are provided.

#### 3.4.3 Surface Phase Diagrams under CO_2_/CO Conditions

The interaction between CO_2_ and CO with the CuWO_4_ surfaces can be described by the following chemical reactions
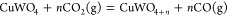
16
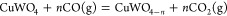
17

The surface free energies of the partially oxidized (σ_oxi_(*T*,*p*)) and partially reduced (σ_red_(*T*,*p*)) CuWO_4_ surfaces under the conditions for CO_2_ reduction and CO oxidation are determined as follows
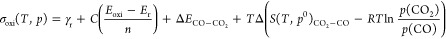
18
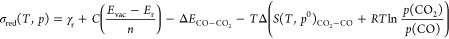
19where  is the logarithm of ratio of the partial pressure of CO_2_ and CO, Δ*E*_CO–CO_2__ denotes the energy difference between CO and CO_2_ from the DFT calculations, and Δ(*S*(*T*,*p*^0^)_CO_2_–CO_ is the entropy difference between CO_2_ and CO at 1 bar extracted from the thermodynamic tables.^55^

**Figure 8:**
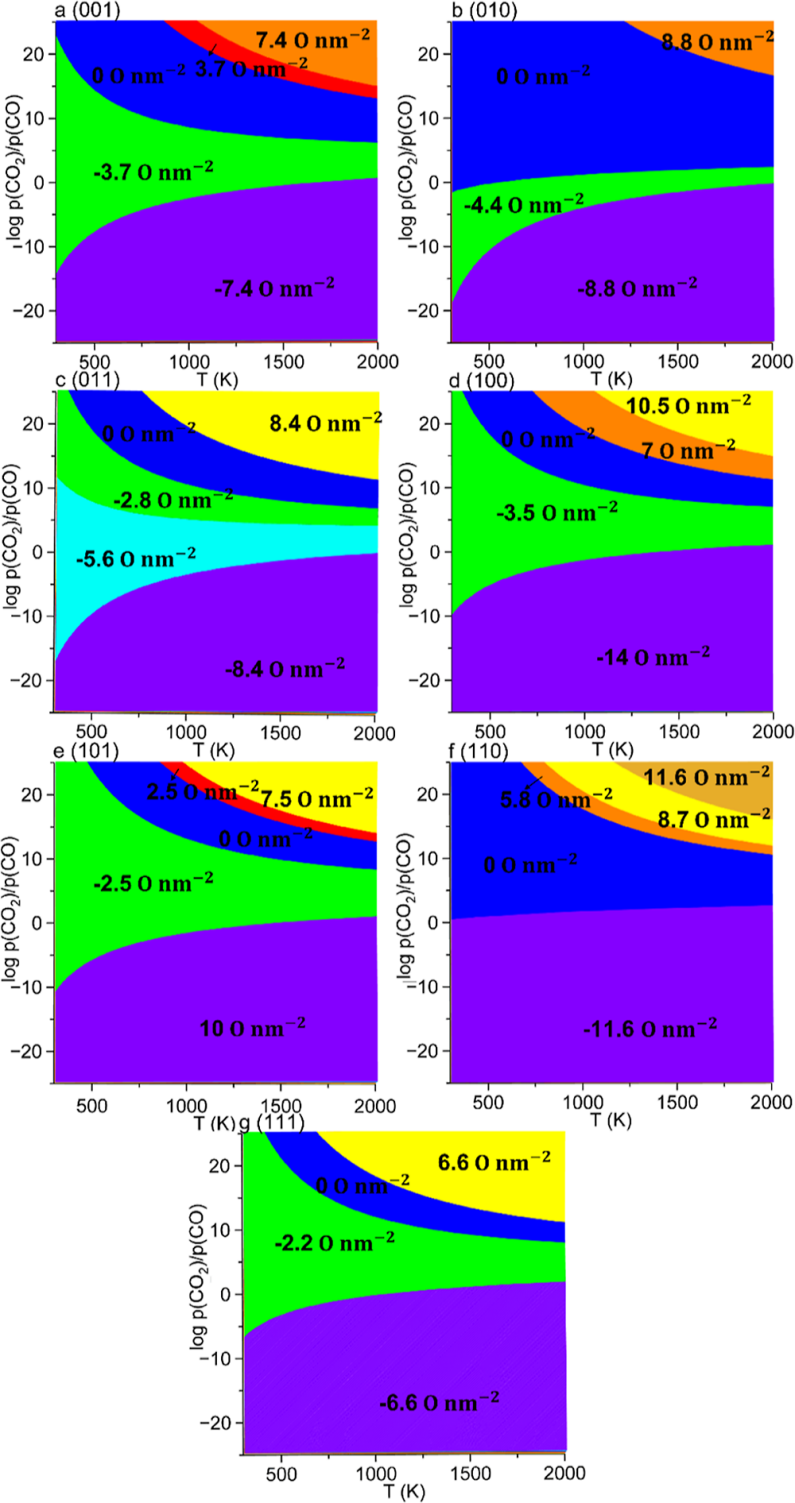
Surface phase diagrams for the low-Miller index surfaces of CuWO_4_ as a function of the logarithm of the ratio of the partial pressures (*p*) of CO_2_/CO and temperature (*T*). The coverages (*C*) of the O adatoms and O vacancies for the nonpristine surfaces are noted in the figures with positive and negative values, respectively.

On the (001) surface, oxidation starts at 900 K when  reaches 15, as indicated by the red region in Figure 8a. The coverage of O adatoms increases to 7.4 O nm^–2^ for a temperature above 1100 K, as depicted by the orange region in Figure 8a. However, we did not find evidence of oxidation when  is below 13. Our calculations suggest that reduction occurs when  is lower than 22 at 298 K, with the surface remaining reduced at a coverage of O vacancies of 3.7 O nm^–2^ when  is between 5 and 1. We found that the surface remains reduced, with a coverage of O vacancies of 7.4 O nm^–2^, when  is below −15, as shown in the purple region of Figure 8a.

Unlike the surface phase diagram under O_2_ conditions, we found that the (010) surface becomes partially oxidized when it is exposed to  = 17 at 1250 K, when the coverage of O adatoms reaches 8.8 O nm^–2^. In contrast, reduction commences when  reaches 3 at 2000 K, resulting in a coverage of O vacancies of 4.4 O nm^–2^, shown in Figure 8b. Notably, the surface remains fully reduced across the entire temperature range from 298 to 2000 K when  falls below 19, corresponding to a coverage of O vacancies of 8.8 O nm^–2^, shown as a purple region in Figure 8b.

On the (011) surface, reduction starts at 298 K with a coverage of O vacancies of 2.8 . Decreasing  to 4 causes the (011) surface to be reduced with a coverage of O vacancies of 5.6 O nm^–2^, corresponding to two oxygen vacancies in our simulation cell. The surface becomes fully reduced with a coverage of O vacancies of 8.4 O nm^–2^ when  is lower than 17.5 at 2000 K, shown as a purple region in Figure 8c. The only level of oxidation, with a coverage of O adatoms of 8.4 O nm^–2^, occurs when the temperature and  are above 750 K and 12, respectively, indicated as a yellow region in Figure 8c.

Similar to the (011) surface, our results suggest that the (100) surface starts to become reduced at 298 K, resulting in a partially reduced surface with a coverage of 3.5 O nm^–2^. When  falls below −10, the surface becomes reduced with a coverage of O vacancies of 14 O nm^–2^ at any temperature between 298 and 2000 K, corresponding to four oxygen vacancies in our simulation cell, shown as a purple region in Figure 8d. We found that oxidation with a coverage of O adatoms of 7 O nm^–2^ occurs when the temperature and  are above 750 K and 12, respectively. Increasing the temperature to 1100 K and  to 5 fully oxidizes the (100) surface with a coverage of O adatoms of 10.5 O nm^–2^, corresponding to the adsorption of three oxygen atoms in our simulation cell, illustrated as a yellow region in Figure 8d.

Reduction of the (101) surface is observed at 298 K with a coverage of O vacancies of 2.5 nm^–2^, shown as a green region in Figure 8e. When  is below −11, the surface becomes fully reduced at any temperature between 298 and 2000 K with a coverage of O vacancies of 10 O nm^–2^, corresponding to four oxygen vacancies in our simulation cell. Oxidation occurs when the temperature and  are above 900 K and 13, respectively. When the temperature is increased above 1000 K and  is above 14, the surface is completely oxidized with a coverage of O adatoms of 7.5 O nm^–2^, corresponding to the adsorption of three oxygen atoms in our simulation cell, shown as a yellow region in Figure 8e.

Our result suggests that the (110) surface can only be reduced with a coverage of O vacancies of 10 O nm^–2^ when  is below 0. The (110) surface starts oxidizing when  increases above 11, resulting in a partially oxidized surface with a coverage of O adatoms of 5.8 O nm^–2^, corresponding to the adsorption of two oxygen atoms in our simulation cell. Increasing the temperature to 800 K and  to 12 partially oxidizes the (110) surface with a coverage of O adatoms of 8.7 O nm^–2^. Further increasing the temperature to 1200 K and  to 16 fully oxidizes the surface with a coverage of O adatoms of 11.6 O nm^–2^, corresponding to the adsorption of four oxygen atoms in our simulation cell, depicted as a dark yellow region in Figure 8f.

Similar to the (110) surface, we found that the (111) facet is partially reduced at 298 K, resulting in a coverage of O vacancies of 2.2 O nm^–2^, shown as a green region in Figure 8g. Decreasing  below −7 increases the coverage of O vacancies to 6.6 O nm^–2^, indicating complete reduction after our simulation slab loses three oxygen atoms. Only one level of oxidation with a coverage of O adatoms of 6.6 O nm^–2^ occurs when the temperature and  are larger than 700 K and 11, respectively, depicted as a yellow region in Figure 8g.

Our computational surface phase diagrams indicate that the CO_2_/CO conditions facilitate the reduction of the material, as CO_2_ is more stable than CO and the latter is a reducing agent. Interestingly, all seven low-Miller index surfaces demonstrate the ability to be fully reduced across the entire temperature range between 298 and 2000 K, provided that certain ratios of the partial pressure of CO_2_ and CO are maintained. Apart from the (010) and (110) surfaces, we found that the remaining facets require  to be below 1 to become reduced. Although we found evidence of oxidation for each surface, our calculations show that it is difficult to oxidize the CuWO_4_ surfaces under CO_2_/CO conditions owing to the large ratio of the partial pressures of CO_2_ and CO required and the low concentration of CO present in the atmosphere.

### 3.5 Morphology of CuWO_4_

The morphologies of the CuWO_4_ nanocrystals in different environments, that is, under synthesis conditions,^57–61^ the conditions for carbon dioxide reduction,^28,62,63^ and the conditions for water splitting,^64–66^ were determined based on the surface phase diagrams discussed in the previous sections. The Wulff morphologies were constructed using the atomic configuration with the lowest surface free energy for each surface, using the following relationships , where σ and *d* represent the surface free energy and the distance from the center to the surface of the crystal.

**Figure 9:**
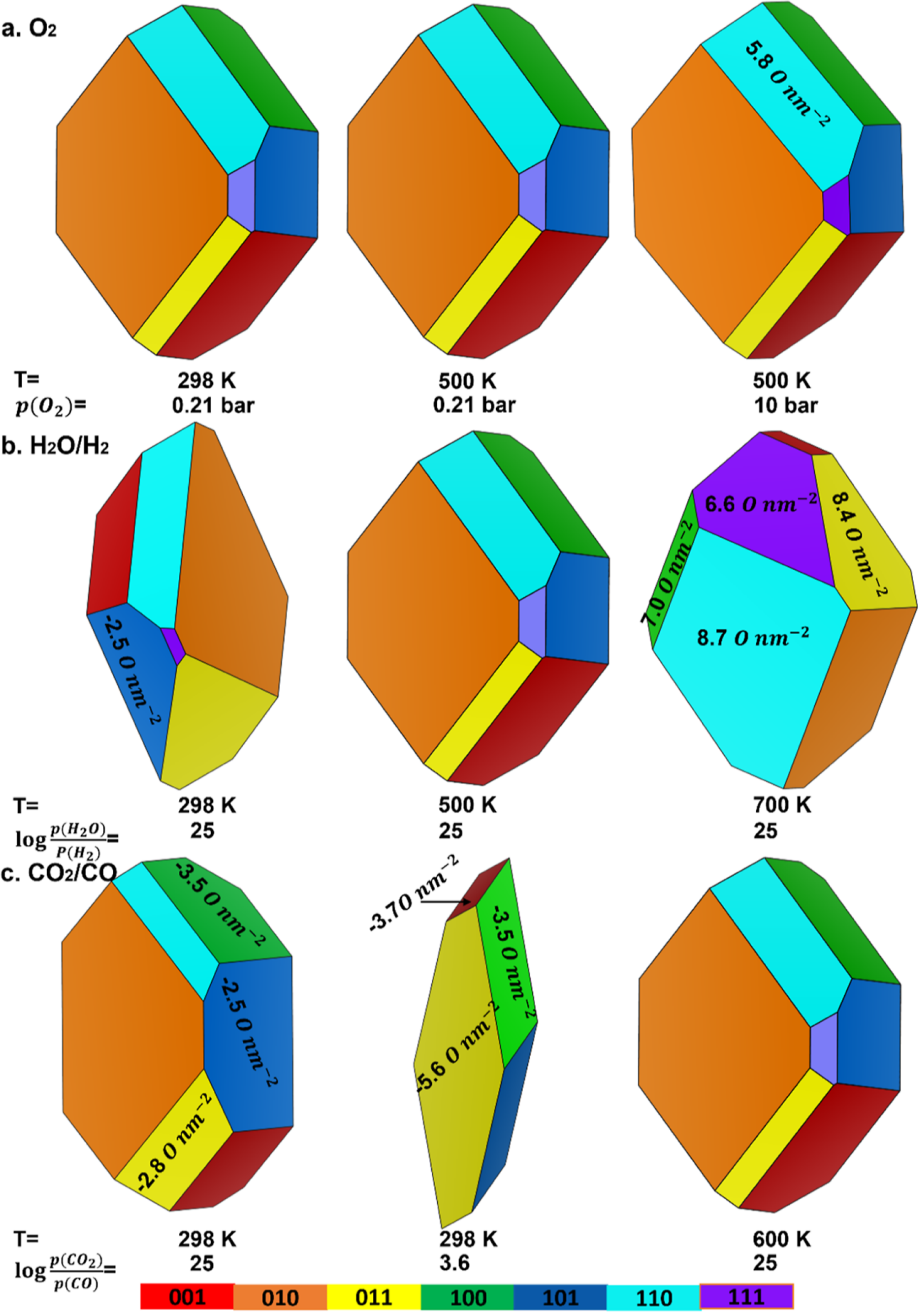
Morphology of the CuWO_4_ nanocrystals (a) under room temperature/pressure, synthesis, and O_2_-rich conditions; (b) in a H_2_O/H_2_ environment under the conditions of water splitting; and (c) in a CO_2_/CO environment under the conditions of carbon dioxide reduction. The coverages of the O adatoms and O vacancies for the nonpristine surfaces are noted in the figures by positive and negative values, respectively.

Figure 9a illustrates that the morphology of CuWO_4_ under typical synthesis conditions (*T* = 500 K and *p*(O_2_) = 0.21 bar) has a tetra-decahedral shape with 10 irregular pentagons and 4 trapezoids. We found that all of the CuWO_4_ surfaces remain pristine up to 500 K, showing significant stability when exposed to environmental O_2_ conditions. The (010) surface is the predominant facet in all of our morphologies under O_2_, with the lowest surface free energy of 36 meV/Å^2^. Our results are consistent with experimental observations under synthesis conditions. Increasing the partial pressure of O_2_ to 10 bar enhances the expression of the partially oxidized (110) surface in the crystal morphology owing to the small surface free energy of this facet with a coverage of O adatoms of 5.8 O nm^–2^. Otherwise, the morphology at *p*(O_2_) = 10 bar exhibits only slight changes compared to that reported under synthesis conditions, corroborating the robust stability of CuWO_4_ in high-temperature and O_2_-rich environments.

To calculate the equilibrium morphology of the CuWO_4_ nanocrystals under the conditions of gas-phase water splitting, we employed  of 25 to simulate the working environment of the photocatalyst under a pure water steam. At a temperature of 298 K, we found that CuWO_4_ transforms into a dodecahedron shape with three pentagons and three trapezoids, as shown in Figure 9b. The partial reduction of the (101) surface with a coverage of the O vacancies of 2.5 nm^–2^ prevents the expression of the (100) surface, resulting in a morphological distortion. When the temperature is increased to 500 K, the morphology returns to the tetradecahedron, similar to the shape exhibited by CuWO_4_ under the synthesis conditions. Further temperature elevation above 700 K leads to oxidation, which lowers the surface free energies of the (011), (100), (110), and (111) surfaces. As a result, the morphology shifts to a dodecahedron dominated by the (110) surface, indicating that CuWO_4_ remains stable up to 500 K in the presence of a pure water steam, as shown in Figure 9b. However, further research is necessary to determine the optimal conditions of temperature and water pressure required for the application of CuWO_4_ as a photocatalyst for the gas-phase water splitting reaction.

We chose a temperature of 298 K to simulate the effect of the CO_2_ conversion reaction at room temperature on the morphology of CuWO_4_.  of 25 and 3.6 were employed to represent pure CO_2_ conditions and ambient conditions, that is, 0.04% CO_2_ and 100 ppb CO in air, respectively. Due to the low surface free energies of the partially reduced (011), (100), and (101) surfaces, as well as the pristine (010) and (110) surfaces at 298 K, the morphology of CuWO_4_ under  = 25 adopts a dodecahedral shape dominated by the (010) surface, featuring two trapezoids, two pentagons, and eight hexagons, as shown in Figure 9c. As  approaches ambient conditions, the morphology shifts to an octahedron with two hexagons and four quadrilaterals. Now, the (011) surface dominates the crystal morphology with a surface free energy of 6 meV/^2^ and a coverage of O vacancies of 5.6 O nm^–2^, whereas the (010), (110), and (111) surfaces are not observed due to their relatively high surface free energies. We did not find evidence of changes in the morphology of CuWO_4_ when the temperature exceeds 600 K at large  values. However, the optimal conditions for CO_2_ conversion at the surfaces of our catalyst require further study.

## 4 Conclusions

In this work, we have reported a computational study based on the DFT of the redox properties of the low-Miller index surfaces of CuWO_4_ and the impact on the crystal morphology of various industrially relevant conditions. First, we simulated the structural and electronic properties of the bulk phase of CuWO_4_, and we found that the lattice parameters, atomic Bader charges, and magnetic moments are in agreement with published works. We have used the optimized bulk structure to create the two nonpolar terminations of each of the seven low-Miller index surfaces and found that the slabs that suffered only minor structural changes during relaxation, or were terminated by Cu rather than W, are usually the thermodynamically most stable. For example, the (010) surface has the smallest relaxed surface energy reported in this study, and the (111) surface is the chemically most reactive surface with the lowest work function. We also investigated the redox properties of each surface and found that the atomic Bader charges undergo negligible change during oxidation, whereas they decrease for each level of (partial) reduction. The magnetic moments of the Cu and W ions change by less than 0.1 μ_B_/atom during oxidation, but they undergo significant changes during reduction due to severe structural distortions of the surfaces. With the exception of the (100) surface, the surface free energies of all of the low-Miller index surfaces of CuWO_4_ are below 0.5 eV/^2^, suggesting that they are thermodynamically stable facets.

The redox surface phase diagrams are reported as a function of the partial pressure of oxygenated species and temperature under three different conditions, that is, O_2_ at ambient and under synthesis conditions, as well as mixtures of H_2_O/H_2_ under the conditions of the gas-phase water splitting reaction and CO_2_/CO under the conditions of CO_2_ reduction. We found that both oxidation and reduction processes of the surfaces occur more readily at relatively high temperatures. The (100), (110), and (111) surfaces are more easily oxidized compared to the rest of the low-Miller index planes under O_2_ conditions, but we did not find evidence of oxidation of the (010) surface at O_2_ pressures below 25 bar. Our surface phase diagrams suggest that a wider range of ratios of partial pressures of H_2_O/H_2_ and CO_2_/CO stabilize the partially reduced surfaces than the partial pressure of O_2_ since H_2_ and CO are strong reducing agents. The (001), (011), (100), (101), and (111) surfaces can become partially reduced at room temperature under a mixture of H_2_O and H_2_, whereas the (011), (100), (101), and (111) surfaces also lose O atoms at room temperature under a mixture of CO_2_ and CO. The pristine (010) surface is the thermodynamically most stable facet, within the widest range of conditions of the three different environments considered in this work, which is consistent with its lowest relaxed surface free energy. Our simulations suggest that the pristine (111) surface, which has the narrowest range of stability conditions, is chemically the most reactive surface owing to its consistently smallest work function at each coverage of O vacancies.

The equilibrium crystal morphology of CuWO_4_ under the three environments was constructed using the surface free energy values from the surface phase diagram corresponding to specific temperatures and ratios of partial pressures of oxygenated species. Our results show that the CuWO_4_ crystals are stable under ambient conditions, whereas the morphology does not change at the typical partial pressure of a pure water steam at least up to 500 K. However, we found that the morphology clearly changes at 298 K under very large partial pressures of CO_2_/CO, consistent with a high purity of CO_2_, and at the same ratio of partial pressures of CO_2_ and CO existing in air. However, the morphology of the CuWO_4_ crystals remains stable up to 600 K in the presence of pure CO_2_.

This study is focused on (i) developing the computational settings to describe the electronic and structural properties of the bulk phase of CuWO_4_ and benchmarking those predictions against previous reports, (ii) modeling the physiochemical properties of all nonpolar and symmetric stoichiometric terminations of the low-Miller index surfaces, and (iii) investigating the redox surface phase diagrams as a function of temperature and the ratio of the partial pressures of oxygenated species under synthesis conditions and in environments rich in H_2_ and H_2_O as well as CO and CO_2_, which are relevant to the photocatalytic water splitting and CO_2_ conversion reactions. Future work will focus on simulating explicitly the gas-phase water splitting and CO_2_ reduction processes under realistic reaction conditions using the most prominent surfaces displayed in the crystal morphologies.

## Supporting Information Available

3D phase diagram of the (010) surface, 2D phase diagram of the (010) surface, and entropy of H_2_O and H_2_ as a function of temperature (PDF)
