# Supplementary material for: Exploring the Redox Properties of the Low-Miller Index Surfaces of Copper Tungstate (CuWO4): Evaluating the Impact of the Environmental Conditions on the Water Splitting and Carbon Dioxide Reduction Processes
Source: J Phys Chem C Nanomater Interfaces. 2023 Sep 15;127(38):18944–61. doi: 10.1021/acs.jpcc.3c04413 (PMC10544046; doi:10.1021/acs.jpcc.3c04413)
Supplement: Supplementary file 1 — jp3c04413_si_001.pdf [file jp3c04413_si_001.pdf]

Supporting information for:

Exploring the Redox Properties of the Low-Miller Index Surfaces of Copper Tungstate ($CuWO_4$): Evaluating the Impact of the Environmental Conditions on the Water Splitting and Carbon Dioxide Reduction Processes

Xuan Chu,[1] David Santos-Carballal,[1,*] Nora H de Leeuw[1,2]

[1]School of Chemistry, University of Leeds, Leeds LS2 9JT, United Kingdom

[2]Department of Earth Sciences, Utrecht University, Princetonplein 8A, 3584 CD Utrecht, The Netherlands

Table of Contents

- Bird's eye view of the non-planar potential energy surface representations of the surface free energies for all the coverages of O, considered for the (010) surface.
- Surface phase diagrams for the low-Miller index (010) surfaces of $CuWO_4$.
- The comparation of the entropy of H_2O (a) and H_2 (b) calculated using statistical thermodynamic and experimental values at different temperature.

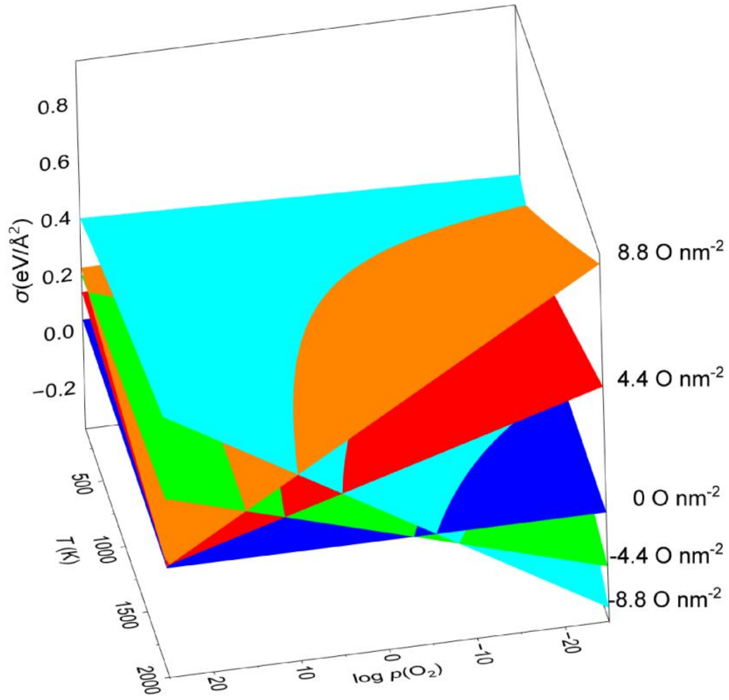

Figure S1. Bird's eye view of the non-planar potential energy surface representations of the surface free energies for all the coverages of O, considered for the (010) surface.

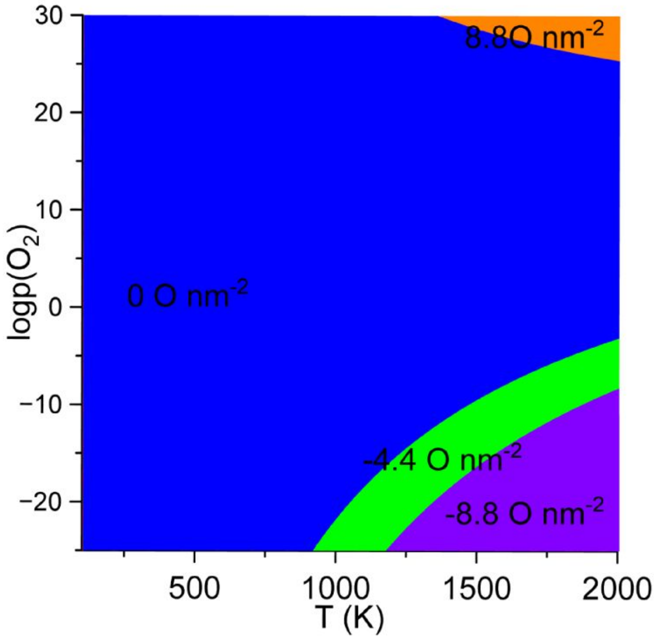

Figure S2. Surface phase diagrams for the low-Miller index (010) surfaces of $CuWO_4$ as a function of the logarithm of the partial pressure of oxygen and temperature. The coverages (*C*) of O adatoms and vacancies with positive and negative values respectively, are indicated for each region.

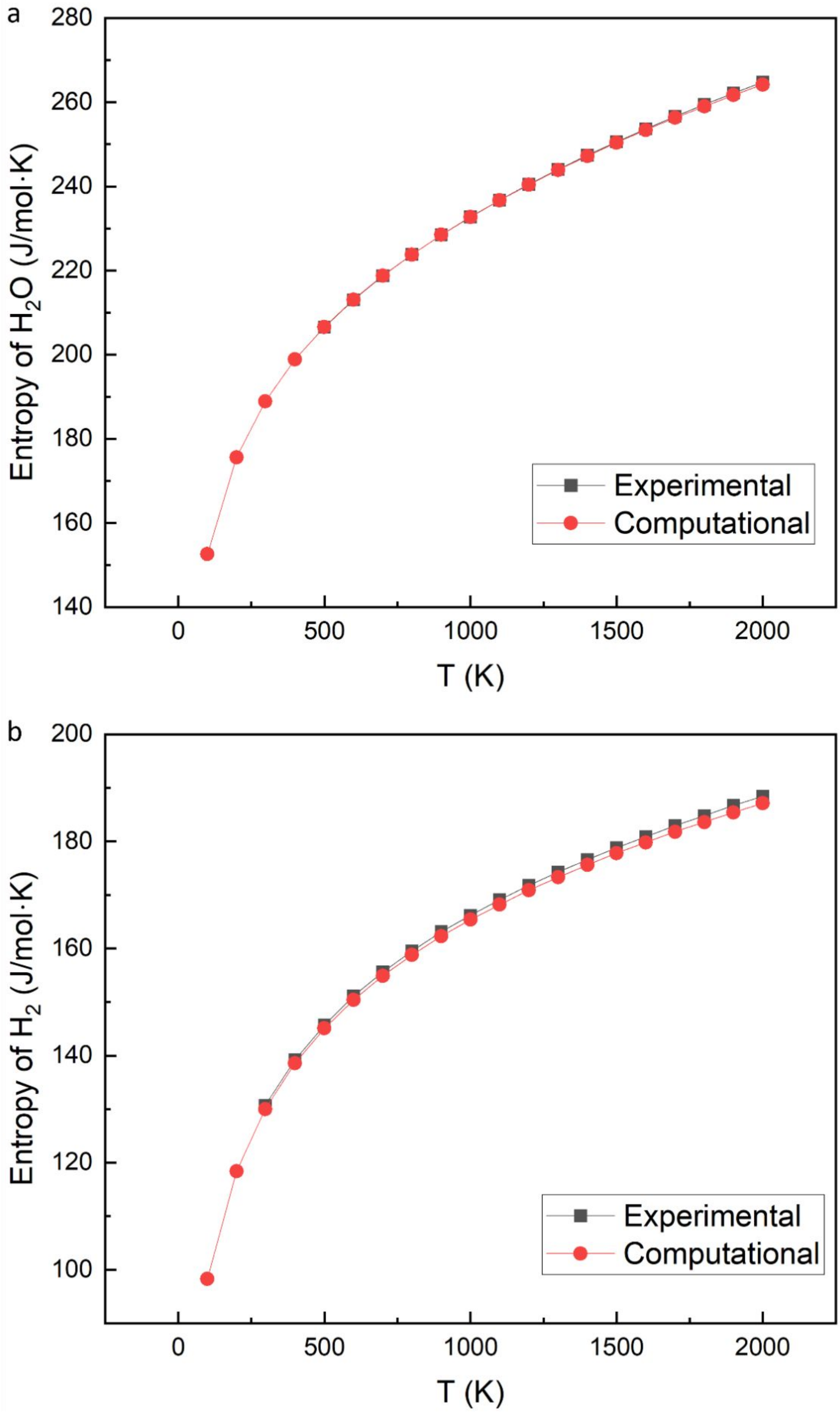

Figure S3. The comparation of the entropy of H_2O (a) and H_2 (b) calculated using statistical thermodynamic and experimental values at different temperature.
